# Boosting the Nucleophilicity of the Diphenylphosphide Anion with Crown Ether Supported Heavy Alkali Metals to Facilitate Highly Efficient Catalytic Alkene Isomerisation

**DOI:** 10.1002/anie.202523460

**Published:** 2026-01-20

**Authors:** Felix Krämer, Thomas M. Horsley Downie, Robert E. Mulvey

**Affiliations:** ^1^ Department of Pure and Applied Chemistry University of Strathclyde Glasgow G1 1XL UK

**Keywords:** Alkali metals, Alkene isomerisation, Alkyne isomerisation, Catalysis, Remote functionalisation

## Abstract

Caesium and rubidium have long experienced an interest drought in organoelement chemistry in comparison to the vast ocean of applications accomplished mainly by lithium and to a lesser extent, by sodium and potassium in this field. Here, we report a breakthrough study in catalytic alkene isomerisation using crown ether‐supported alkali metal phosphides in which the activity increases sequentially and significantly as Group One is descended with Cs(18‐crown‐6)PPh_2_ performing best even at 1 mol% loadings with high turnover frequencies (TOFs) and good functional group tolerance. Elevating its profile further, Cs(18‐crown‐6)PPh_2_ is also successful in a stepwise double catalysis on combining alkene isomerisation with its newly established hydrophosphination (HP) capacity to access challenging to make Markovnikov products. This remote functionalisation approach was also applied to alkynes with terminal alkynes preferentially producing allenes over internal alkynes in one‐pot reactions and then under HP, generating highly functionalised vinyl phosphines. Kinetic studies and DFT calculations have also been performed to shed light on mechanistic aspects of the Cs‐mediated isomerisation of model alkene allylbenzene.

## Graphical abstract

Here, we report a breakthrough study in catalytic alkene isomerisation using crown ether‐supported alkali metal phosphides in which the activity increases sequentially and significantly as Group One is descended with Cs(18‐crown‐6)PPh_2_ (**1^Cs^**) performing best. Rocketing its profile further, **1^Cs^** is also successful in remote functionalisation of alkenes and alkynes by a stepwise double catalysis on combining alkene isomerisation with its newly established hydrophosphination (HP) capacity to access challenging to make Markovnikov products.

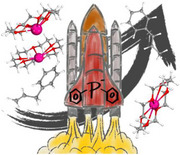

## Introduction

Alkenes are simple and essential building blocks in chemistry, biology and materials science.^[^ ^1^, ^2^, ^3^, ^4^ ^]^ While the cracking of alkanes^[^ ^5^, ^6^ ^]^ and the shell process to produce higher olefins (SHOP)^[^ ^7^ ^]^ yield terminal alkenes, the dehydrogenation of alkanes yields mixtures of both internal and terminal alkanes.^[^ ^8^ ^]^ Internal alkenes are accessible by various methods such as dehydrohalogenation of alkyl halides or dehydration of alcohols^[^ ^9^ ^]^ as well as elimination of amines,^[^ ^10^ ^]^ carbonyl olefination,^[^ ^11^, ^12^, ^13^, ^14^, ^15^ ^]^ olefin metathesis,^[^ ^16^ ^]^ partial hydrogenation of alkynes,^[^ ^17^ ^]^ and dehydro cross‐coupling.^[^ ^18^ ^]^ Catalytic alkene isomerisation offers an outstanding atom‐economical alternative of growing importance in this area.^[^ ^19^, ^20^, ^21^ ^]^ Innovative approaches in which further functionalisation follows the isomerisation offer a simple way to access higher value, chemically more complex products, emphasising the importance of such transformations.^[^ ^22^, ^23^, ^24^, ^25^, ^26^, ^27^, ^28^ ^]^

**Scheme 1:**
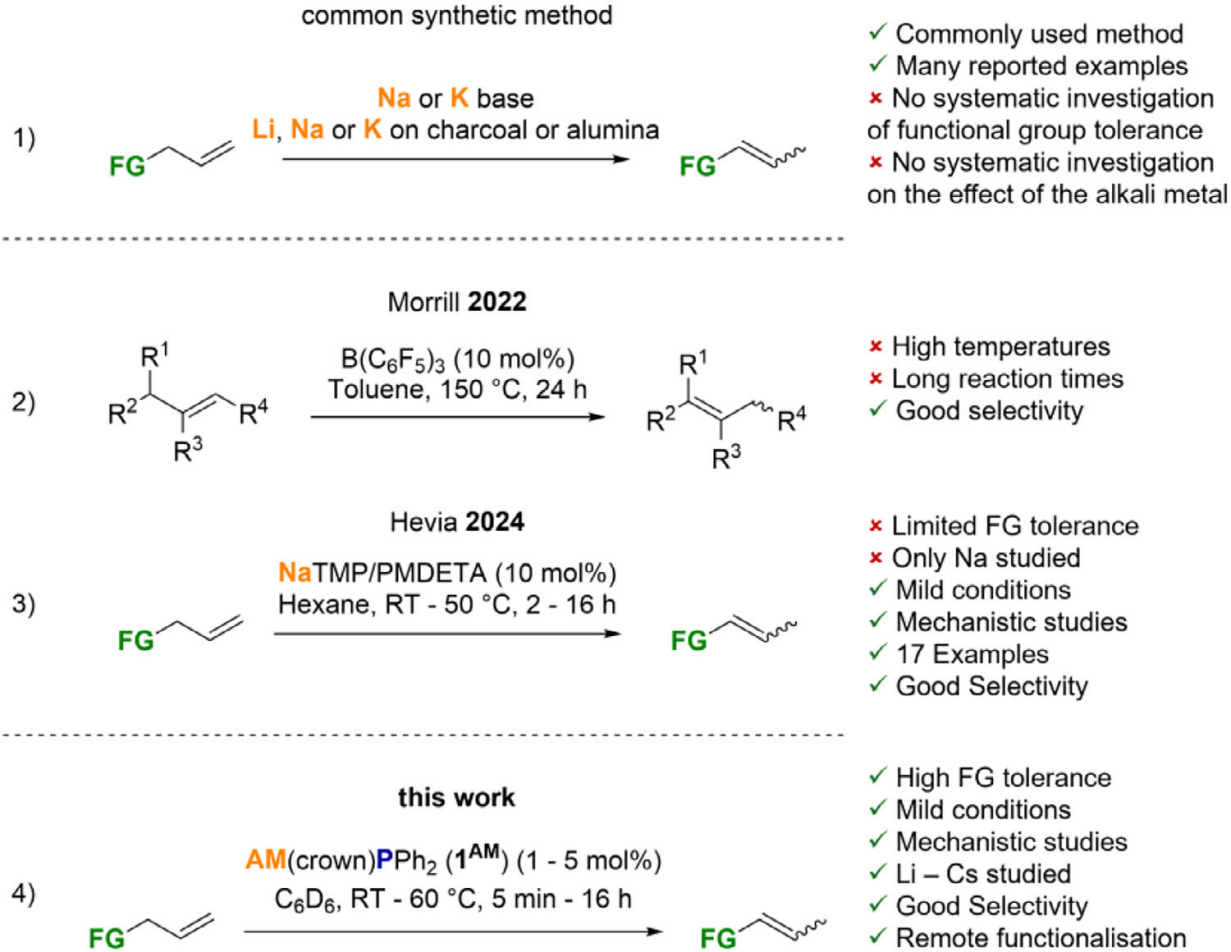
Published main group element compound catalysed alkene isomerisation reactions. AM(crown) = Li(15‐crown‐5), Na(15‐crown‐5), K(18‐crown‐6), Rb(18‐crown‐6), Cs(18‐crown‐6).

While both precious and abundant transition metal‐based catalysts dominate the field of catalytic alkene isomerisation, by comparison, main group element‐based systems are greatly underrepresented.^[^ ^29^, ^30^, ^31^, ^32^, ^33^, ^34^, ^35^ ^]^ In one of the few reported examples, the popular, versatile Lewis acid B(C_6_F_5_)_3_ catalyses the isomerisation of alkenes, but high temperatures (140 °C–150 °C) and long reaction times of 24–48 h are required (Scheme 1).^[^ ^36^, ^37^, ^38^ ^]^ Alkali metal Brønsted bases are predestined for such transformations. Using alkoxides or small amides such as LDA (lithium diisopropylamide, LiN*^i^* Pr_2_) the transformation stays within the limits of stoichiometric reactions.^[^ ^19^, ^39^, ^40^, ^41^, ^42^, ^43^, ^44^, ^45^ ^]^ On the other hand, potassium or sodium amides, alkoxides and alkyl derivatives, as well as alkali metals on charcoal or alumina, are reported as effective catalysts for alkene and alkyne isomerisation mostly using polar solvents representing a well‐studied method of synthetic chemistry turning terminal alkenes into the thermodynamically more stable internal alkenes.^[^ ^46^, ^47^, ^48^, ^49^, ^50^, ^51^, ^52^ ^]^ Although many reports of the named transformation exist, few systematic studies about the functional group tolerance of a specific catalytic system are presented. Furthermore, no systematic study showcasing the dependency of the alkali metal, especially the effect of the heaviest ones, has been reported yet. Recently, Hevia and coworkers published a seminal study in which they used the superbasic NaTMP (TMP = 2,2,6,6‐tetramethylpiperidide) partnered with PMDETA (*N,N,N′,N″,N″*‐pentamethyldiethylenetriamine) as a ligand to isomerise a range of activated and non‐activated substrates (Scheme 1).^[^ ^53^ ^]^ This represented the first systematic investigation of the catalytic activity of sodium bases in the title reaction class and proved by a combination of experimental and quantum chemical analysis that the isomerisation in their case proceeds via allylic metalation of the substrates. However, the phenomenal high reactivity of NaTMP prevented the use of carbonyl‐, nitrile‐ or halogen‐substituted substrates.

During work on the catalytic activity of our recently presented crown ether supported alkali metal phosphides of general formula AM(crown)PPh_2_ [**1^AM^**: AM(crown) = Li(15‐crown‐5),^[^ ^54^ ^]^ Na(15‐crown‐5),^[^ ^55^ ^]^ K(18‐crown‐6),^[^ ^56^ ^]^ Rb(18‐crown‐6),^[^ ^54^ ^]^ Cs(18‐crown‐6)^[^ ^54^ ^]^] in the hydrophosphination (abbreviated throughout as HP) of unsaturated hydrocarbons, we found that **1^Cs^** isomerises allylbenzene **2** to *β*‐methyl‐styrene which is subsequently hydrophosphinated.^[^ ^57^ ^]^ This led to the formal Markovnikov product, which is generally more difficult to access.^[^ ^58^ ^]^ Inspired by this observation, combined with the increasing number of examples in recent times in which the heavy alkali metals, in particular K, Rb, and Cs, outperform the reactivity and catalytic activity of their lighter congeners Li and Na by orders of magnitude, it was of interest and urgency to us to ascertain how far we could push the limits of this important class of reaction by using heavy alkali metal bases.^[^ ^57^, ^59^, ^60^, ^61^ ^]^ Herein, we present the intriguing results of a comparative study investigating the activity of the complete set of **1^AM^** (AM = Li–Cs) complexes in the catalytic isomerisation of alkenes (Scheme 1) and the potential of **1^Cs^** for remote functionalisation by isomerisation‐hydrophosphination (HP) double catalysis.

## Results and Discussion

### Catalyst Screening

**Scheme 2:**
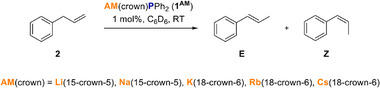
Conditions of catalytic isomerisation of alkene **2** using 1 mol% **1^AM^** as the catalyst.

As a benchmark system, we chose the aforementioned isomerisation of allylbenzene **2** (Scheme 2). First, we tested the whole set of **1^AM^** Li–Cs in C_6_D_6_ from which it became obvious that **1^Cs^** shows by far the best performance (results are summarised in Table 1).

**Table 1:** Reaction times, conversions and TOFs for catalytic isomerisation of **2** at RT using 1 mol% **1^AM^** as catalyst.

		C_6_D_6_			MeCN‐d_3_		
Entries	Catalysts	Time/min	Yield/% (E:Z)	TOF/h^−1^	Time/min	Yield/% (E:Z)	TOF/h^−1^
1	**1^Li^**	24 h	<1	−	−	−	−
2	**1^Na^**	24 h	25 (15:1)	1	520	>99 (23:1)	11
3	**1^K^**	30	>99 (12:1)	201	15	>99 (28:1)	389
4	**1^Rb^**	23	>99 (11:1)	265	18	>99 (27:1)	329
5	**1^Cs^**	12	>99 (11:1)	493	15	>99 (25:1)	382
6	**1^nBu4N^**	24	<1	−	−	−	−

While the reaction with **1^Cs^** as a catalyst was complete after only 12 min, the rubidium and potassium congeners required 23 and 30 min, respectively. Removal of the alkali metal using the ammonium derivative [*^n^* Bu_4_N][PPh_2_] (**1^nBuN4^**) in its place causes the reaction to cease completely, thus establishing *alkali metal mediation*.^[^ ^62^ ^]^ The activity change is much more substantial than that from Cs to K, when using the lighter alkali metals Li and Na since **1^Li^** showed no conversion at all and **1^Na^** reached only 25% after 24 h. This trend is matched by the turnover frequencies (TOFs) of 493 h^−1^ (**1^Cs^**), compared to 265 h^−1^ (**1^Rb^**), 201 h^−1^ (**1^K^**) and only 1 h^−1^ (**1^Na^**), which decrease appreciably on ascending the group. This reflects very well the trend we have recently observed in the alkali metal‐mediated HP of 1,1‐diphenylethylene (1,1‐DPE). The reaction proceeds with excellent selectivity in all cases with a ratio of the two possible product isomers (***E***: ***Z***) of 11:1 (25:1 in MeCN) for Cs, 11:1 (27:1 in MeCN) for Rb, 12:1 (28:1 in MeCN) for K and 15:1 (23:1 in MeCN) for Na. The lower ***E***: ***Z*** for the heavier alkali metals in benzene‐d_6_ may be due to the fact that the ***Z*** isomer can be seen in higher quantity due to short reaction times, while for long reaction times (Na) the ratios increase in favour of the energetically similar ***E*** isomer. In MeCN‐D_3_ the ***E***: ***Z*** ratios generally increase compared to C_6_D_6_ showing the highest ratio for **1^K^** and the lowest for **1^Na^**. **1** ^**K**^ − **1** ^**Cs**^ even manage to outperform NaTMP/PMDETA (10 mol%) by far in this reaction, which yields 77% after 16 h at RT (97% conversion), resulting in a TOF of 0.5 h^−1^.^[^ ^53^ ^]^ The Lewis acid B(C_6_F_5_)_3_ (10 mol%) requires 24 h at a temperature of 150 °C to achieve 97% conversion (TOF: 0.4 h^−1^).^[^ ^37^ ^]^

We have also observed that the catalytic activity of **1^AM^** in the HP of 1,1‐DPE can be ramped up by increasing the solvent polarity.^[^ ^57^ ^]^ This was evidenced by investigating the isomerisation of **2** in the polar solvent MeCN‐d_3_ and finding that the activity of the heavy alkali metals K–Cs approximately converges with TOFs of 382 h^−1^ (**1^Cs^**), 329 h^−1^ (**1^Rb^**) and 389 h^−1^ (**1^K^**), with the increase in activity being great for K (doubling) but with Cs appearing to be somewhat inhibited. The greatest increase in activity was observed for **1^Na^**, which now reaches full conversion just short of 10 h and the TOF elevenfold to 11 h^−1^.

Yields were determined by NMR integration relative to C_6_Me_6_ or toluene as an internal standard based on the decrease in the substrate signal integration. For 1 mol% catalyst loading, TOFs are calculated from the yield divided by the time required for conversion.

To help towards investigating the origin of this increased activity, we subjected the catalysts to a diffusion ordered spectroscopy (DOSY) NMR study in MeCN‐d_3_ (for more details, see Section S4). In summary, based on the parameters determined, compared with the corresponding DOSY NMR studies in C_6_D_6_ solution^[^ ^54^ ^]^ it can be clearly discerned that the equilibrium between molecular compounds with AM(crown)‐PPh_2_ contact and solvent‐separated ion pairs (SSIPs) shifts more in favour of the SSIPs. This effect is most noticeable for **1^Cs^** and decreases on ascending the group, which is consistent with the decreasing binding energy between the AM(crown) and PPh_2_ (Cs < Rb < K < Na < Li) moieties.^[^ ^54^ ^]^ This trend is also reflected in the ^31^P{^1^H} NMR shifts of **1^AM^**, which all lie between *δ*_31P_ = −3 to −4 ppm in MeCN‐D_3_, whereas in C_6_D_6_ the chemical shift varies significantly [*δ*_1H_ = −17.7 ppm (**1^Na^**), −6.6 (**1^K^**), −3.3 (**1^Rb^**) and +1.5 (**1^Cs^**)]. This indicates an increase in the nucleophilicity of the PPh_2_ anion induced by the weaker interaction with the alkali metal fragment. The comparatively small increase in reactivity of the heaviest alkali metals, Rb and Cs, may be explained by the fact that SSIPs are also present to a certain extent in non‐polar solvents.

### Substrate Screening

**Scheme 3:**
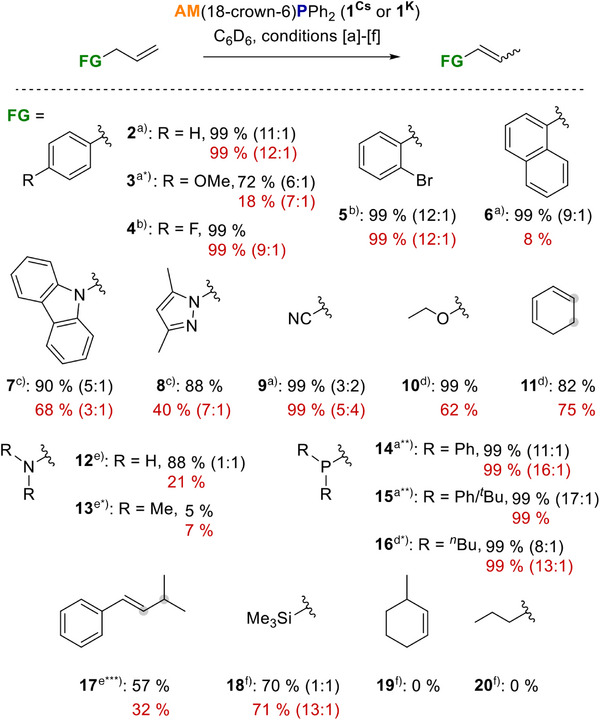
**1^Cs^** catalysed isomerisation of substrates 2–20. Yields and ratios (*E*:*Z*) for Cs (black) and K (red) determined by NMR integration relative to C_6_Me_6_ as an internal standard. ^a)^ 1 mol%, RT, 30 min; ^a*)^: 8 h instead of 30 min; ^a**)^: 1 h instead of 30 min; ^b)^ 5 mol%, RT, 30 min; ^c)^ 5 mol%, RT 4 h; ^d)^ 5 mol%, 60 °C, 18 h; ^d*)^: 3 h instead of 18 h; ^e)^ 5 mol%, 80 °C, 40 h; ^e*^: 4 d instead of 40 h; ^e***)^ 24 h instead of 40 h; ^f)^ MeCN‐d_3_ 5 mol%, 80 °C, 19 h.

Phosphide **1^Cs^** identified as the most efficient catalyst in non‐polar solvents, was then probed for its tolerance towards functional groups. For comparison, all the experiments were paralleled with **1^K^**. Four different conditions were defined in the course of the experiments: [a] 1 mol%, RT, 30 min; [b] 5 mol%, RT, 30 min; [c] 5 mol%, RT, 4 h; [d] 5 mol%, 60 °C, 18 h; [e] 5 mol%, 80 °C, 40 h; [f] MeCN‐d_3_ 5 mol%, 80 °C, 19 h with the results summarised in Scheme 3. **1^Cs^** as well as **1^K^** tolerate aromatic halides (**4**, **5**), nitriles (**9**), N‐heterocycles (**7**, **8**), ethers (**3**, **10**) as well as amines (**12**, **13**) and phosphines (**14**–**16**) as functional groups and thus tolerate a broader spectrum than Hevia's super basic NaTMP/PMDETA system. Furthermore, 1,4‐cyclohexadiene (**11**) can be isomerised to the more stable 1,3‐cyclohexadiene and the more complex 1,1‐dimethyl‐3‐phenylpropene (**17**) is isomerised in satisfactory yields. The non‐activated substrate allyltrimethylsilane (**18**) could be isomerised by **1^Cs^** in MeCN‐d_3_ with satisfactory yields, whereas 3‐methylcyclohexene (**19**) and 1‐hexene (**20**) remained unreacted. Carbonyl functionalities, thioethers and alkyl halides led to the decomposition of **1^Cs^**. Overall the performance of **1^Cs^** is slightly better than that of its potassium congener, yielding higher product quantities with comparable ***E***: ***Z*** ratios.

**Scheme 4:**
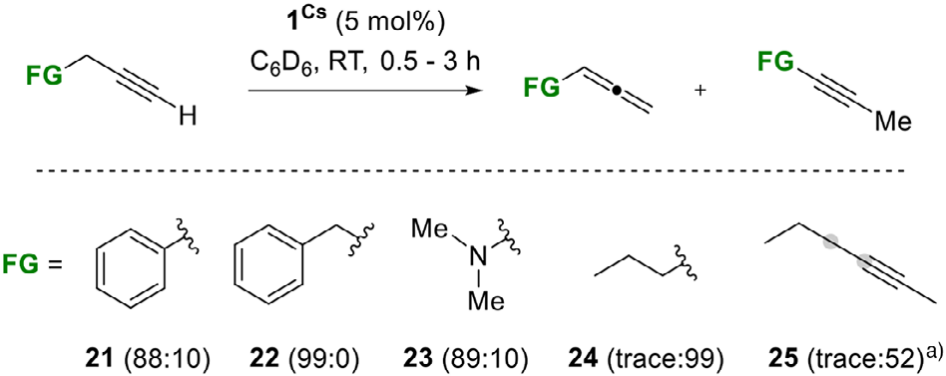
**1^Cs^** catalysed isomerisation of alkynes **21**–**25**. Yields and ratios (allene:internal alkyne) were determined by NMR integration relative to C_6_Me_6_ as an internal standard. ^a)^ 19 h, 80 °C.

When **1^Cs^** (5 mol%) was added to the alkynes **21**–**25** under catalytic conditions, the corresponding allenes were formed in good selectivity (Scheme 4). In these reactions, an equilibrium mixture is usually observed that favours the internal alkyne. Therefore, it is difficult to obtain preparatively useful yields of allenes.^[^ ^63^ ^]^ To our surprise, the reactions stopped at the corresponding allenes and formed only 10% of the internal alkyne for **21** and **23**, whereas **22** gave a 99% yield of allene. No further conversion to the internal alkyne is observed when the reaction mixtures are heated. For 1‐hexyne (**24**), full conversion to the internal alkyne 2‐hexyne was observed within 30 min at RT. The internal 3‐hexyne (**25**) must be heated to 80 °C over 19 h yielding 52% of the isomerised alkyne 2‐hexyne.

### Remote Functionalisation

**Scheme 5:**
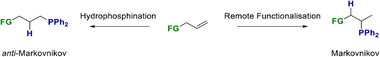
Various products obtained by HP or remote functionalisation.

A major challenge in preparative chemistry is to perform functionalisation of molecules at less reactive positions. Remote functionalisation (RF) is a concept in which an initial interaction with a functional group leads to a selective reaction at a different position of the molecule.^[^ ^23^ ^]^ If we now combine the isomerisation described above with the recently shown HP chemistry, we can obtain the difficult to access Markovnikov products by double catalysis or RF (Scheme 5). This method is facilitated by the phenyl group stabilising the adjacent anionic charge, enabling the HP of the resulting styrene derivatives, where HP of the terminal alkenes is not feasible.^[^ ^64^ ^]^

**Scheme 6:**
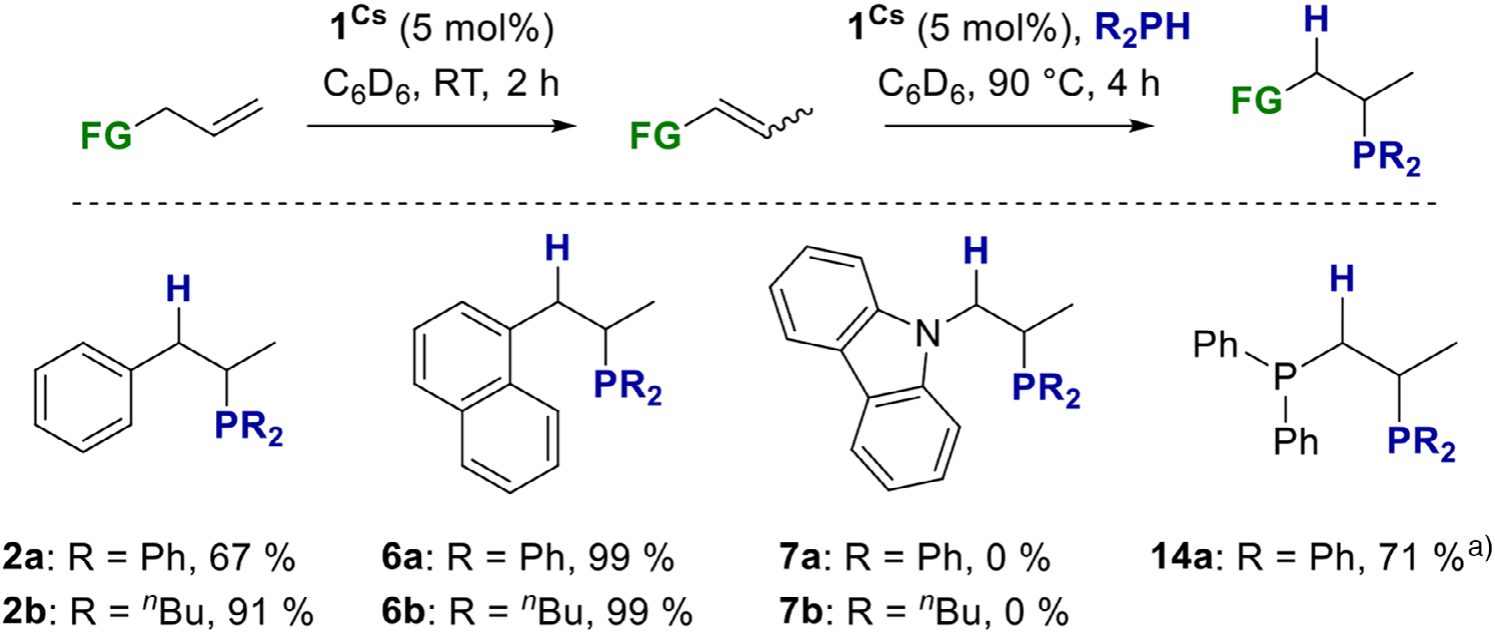
**1^Cs^** catalysed RF of alkenes. ^a)^ 50 h instead of 4 h.

For this purpose, we mixed **1^Cs^** (5 mol%) with substrates **2**–**7** and **14** and added Ph_2_PH (**a**) or *^n^* Bu_2_PH (**b**) after 2 h at RT. Following heating at 90 °C for 4 h, the HP products **2a**, **2b**, **6a**, **6b** and **14a** were obtained in very good yields (Scheme 6). The yields obtained in this way exceed those shown previously, where the phosphine was added directly to the mixture of catalyst and substrate. Alkenes **3** and **4** showed decomposition of **1^Cs^** at elevated temperature, where for the anisole derivative **3**, we were able to characterise the decomposition product of ether cleavage [Cs(18‐crown‐6)‐O‐C_6_H_4_‐(CH)_2_‐CH_3_] using SC‐XRD methods (more details are in Section S6). For the sterically highly demanding substrate carbazolyl derivative **7**, no conversion was observed for either phosphine.

**Scheme 7:**
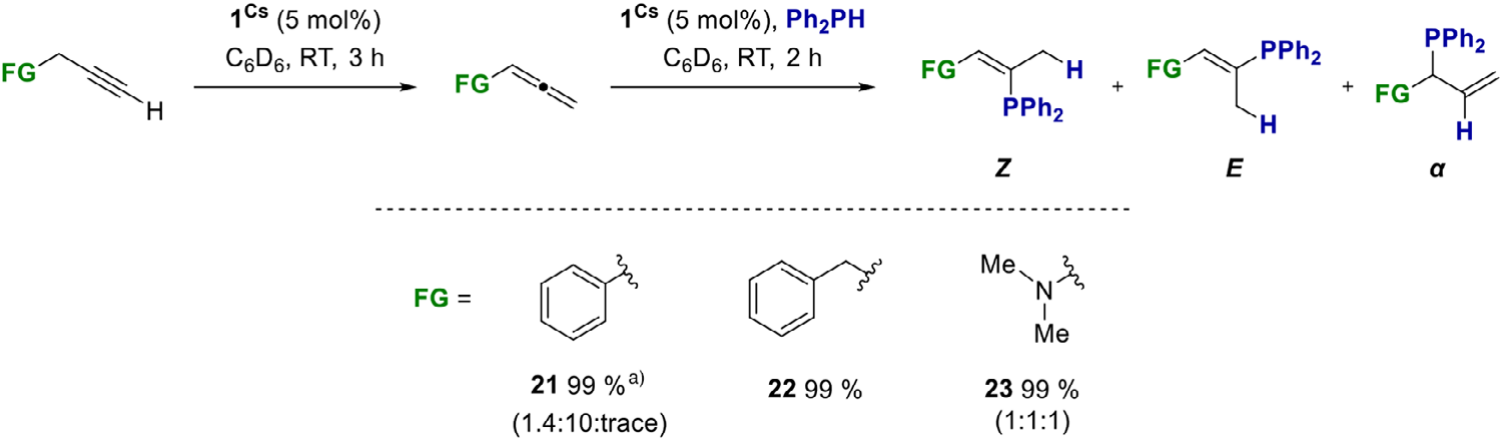
**1^Cs^** catalysed RF of alkynes 21–23. ^a)^ 90 °C 24 h.

We followed the same approach for the RF of alkynes **21**–**23** to generate highly functionalised vinyl phosphines in a one‐pot reaction (Scheme 7). After the **1^Cs^** catalysed isomerisation of **21**–**23** was complete, we added Ph_2_PH to the corresponding reaction mixtures. The HP reactions showed full conversion for **22** and **23** after 2 h at RT. For **22**, the formation of three products was observed, but the product distribution could not be determined due to overlapping signals in the NMR spectra. For **23**, a product distribution (***E***: ***Z***: ***α***) of 1:1:1 was obtained. The HP of **21** required a 90 °C and 24 h reaction time, yielding 99% with a reasonably good selectivity of 1.4:10 (***E***: ***Z***) and only minor traces of the *α*‐product.

### Mechanistic Investigations

In order to gain some insight into the isomerisation reaction mechanism, we first carried out kinetic studies. We used allylanisole **3** and first varied the catalyst concentration at a constant substrate concentration (Figures S125–S128). Next, we varied the substrate concentration while keeping the catalyst concentration constant (Figures S129–S131). These experiments indicated that the reaction is 1^st^ order in catalyst and zero order for the substrate, which is consistent with the reported data.^[^ ^19^, ^65^ ^]^ Product inhibition was not observed in the corresponding experiment (Figure S132). Next, we investigated the isomerisation of **2** at different temperatures for each of the heavier alkali metal sets **1^K^**–**1^Cs^** as catalysts (Figures S133–S135), which provided the experimental data for the activation energies *E*_a_ that are summarised in Table 2. *E*_a_ (in kcal mol^−1^) increases sequentially from **1^Cs^** (11.4) and **1^Rb^** (13.2) to **1^K^** (15.5) by about 2 kcal mol^−1^ which aligns well with the trend in reactivity outlined above.

**Table 2:** Experimentally and computationally (CPCM(benzene)‐TPSSh‐D3BJ/def2‐TZVPP//CPCM(benzene)‐(RI)‐BP86‐D3BJ/def2‐SVP) determined activation energies *E*_a_ and barriers *ΔG*_1_^‡^ for the **1^AM^** catalysed isomerisation of **2**.

	**1^Na^**	**1^K^**	**1^Rb^**	**1^Cs^**
***E*_a(exp.)_/kcal** **mol^−1^**	−	15.5 ± 0.12	13.2 ± 0.60	11.4 ± 0.6
***ΔG*_1_^‡^ (CIP)/kcal** **mol^−1^**	14.6	13.6	18.5	14.9
***ΔG*_1_^‡^ (SSIP)/kcal** **mol^−1^**	6.8	13.9	8.9	6.4

**Figure 1:**
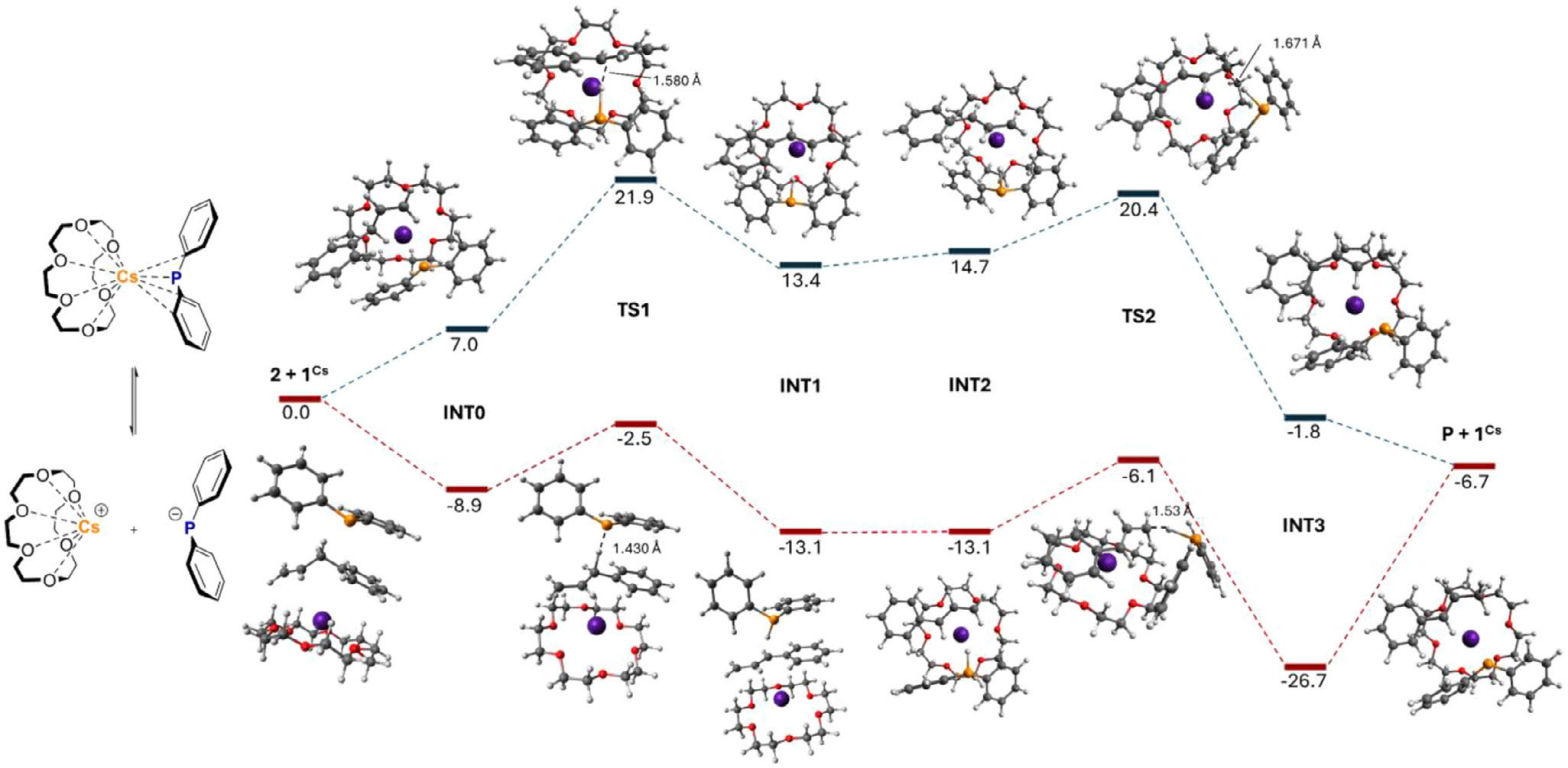
Calculated (CPCM(benzene)‐TPSSh‐D3BJ/def2‐TZVPP//CPCM(benzene)‐(RI)‐BP86‐D3BJ/def2‐SVP) reaction profile for the 1^Cs^ catalysed isomerisation of 2 for both possible starting points (CIPs blue and SSIPs red). Relative free energies (*ΔG*, at 298 K) are given in kcal mol^−^1. For clarity, the molecular models of intermediates and transition states are given chemdraw representations in Scheme 8.

**Scheme 8:**
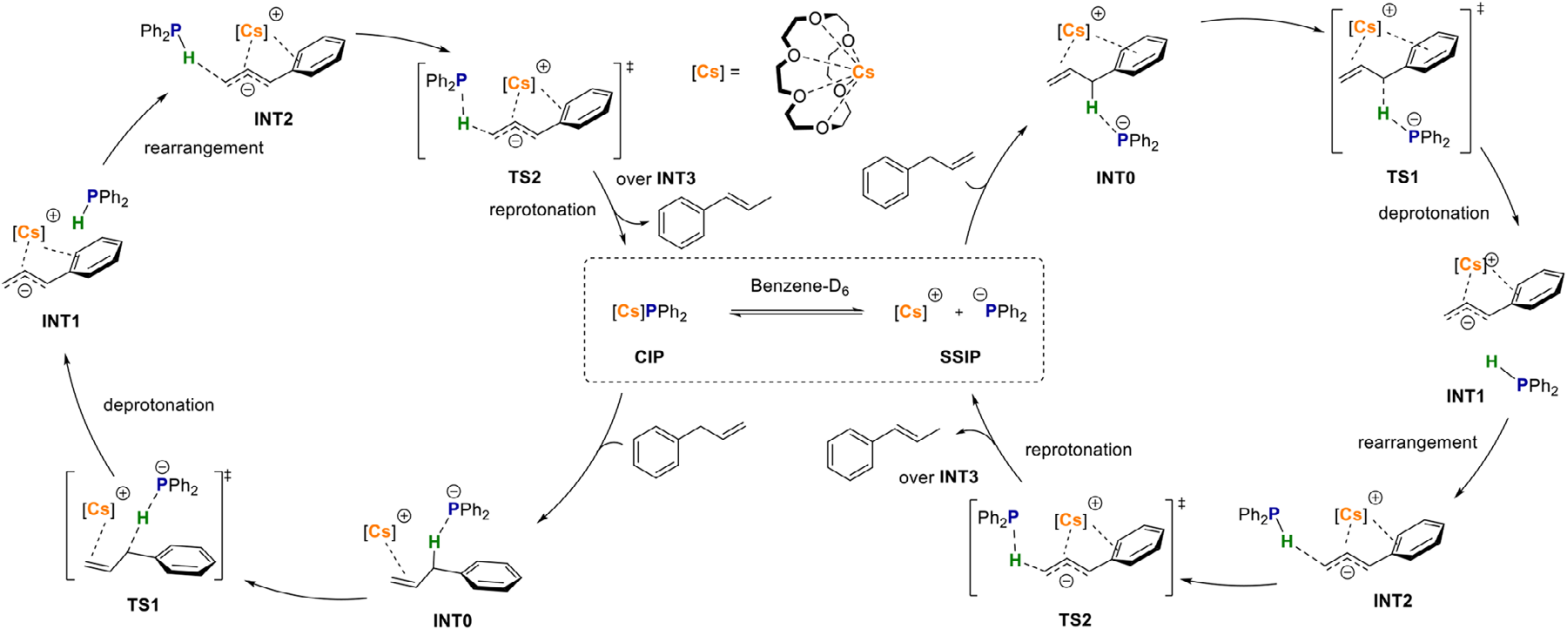
Proposed catalytic cycles of both pathways starting from contact ion pairs (CIP, left) and solvent‐separated ion pairs (SSIP, right).

Furthermore, we calculated the reaction pathway using the CPCM(benzene)‐TPSSh‐D3BJ/def2‐TZVPP//CPCM(benzene)‐(RI)‐BP86‐D3BJ/def2‐SVP level of theory (more details are in Section S7). As can be seen in Figure 1 and Scheme 8, we have chosen two different starting points at which the catalyst is present, on the one hand, molecular with an AM(crown) contact (contact ion pairs, CIPs) and, on the other hand, as SSIPs. Both pathways involve coordination of substrate **2** followed by deprotonation via transition state **TS1**. This is followed by a rearrangement from intermediate **INT1** to **INT2**, in which the phosphine migrates to the terminal carbon atom. Reprotonation via **TS2** with a smaller barrier [5.8 (TS2‐INT2) versus 14.9 (TS1‐INT0) kcal mol^−1^] results in the product complex **INT3**, which in turn decomposes into the catalyst and product. The major difference between these two pathways is that the reaction starting from the CIP proceeds “on top” of the catalyst, while the reaction involving the SSIP proceeds “between” the cation and the anion. The reaction proceeds with an overall energy of −6.7 kcal mol^−1^, which is consistent with reported systems.^[^ ^19^ ^]^

Compared to the experimental activation energies, the calculated values for the barrier (*ΔG*_1_^‡^) of the rate determening step (RDS) of the blue (upper) profile are higher (Table 2), which leads to the assumption that the starting point of the calculations lies between the two cases, Cs(crown)‐PPh_2_ contact and SSIPs, depicted in Figure 1. This is reinforced by the red (lower) calculated profile (Figure 1), which has the SSIPs as its starting point and better reflects the conditions found in the experiment (exceptionally fast reaction at RT). We reason that the catalysts **1^AM^** partly dissociate and then enter the catalytic cycle by providing enough space for the substrate to coordinate. The calculated barriers of the different alkali metals following the red pathway are calculated as 6.4 (**1^Cs^**), 8.9 (**1^Rb^**), 13.9 (**1^K^**) and 6.8 kcal mol^−1^ (**1^Na^**) satisfactorily mirroring the experimental trend in increasing reactivity on descending the group, where **1^Na^** represents an outlier. Note that although the barrier *ΔG*_2_^‡^ for **1^Cs^** is slightly (0.6 kcal mol^−1^) higher with 7 kcal mol^−1^ as *ΔG*_1_^‡^, here the barriers for deprotonation (TS1) are compared. The deprotonation step represents the rate‐determining step, which has been extensively studied for base‐catalysed isomerisation of allylbenzene.^[^ ^19^ ^]^ We recently reported the calculated bond energies between the AM(crown) and PPh_2_ fragments applying an EDA‐NOCV analysis. The bond energy is decreasing [−103.3 (**1^Na^**); −97.5 (**1^K^**); −95.2 (**1^Rb^**); −93.8 kcal mol^−^1 (**1^Cs^**)] on descending the group, which reflects well the experimental trend in the activity and explains why Na shows the lowest activity due to higher energy demand for dissociation into SSIPs.^[^ ^54^ ^]^ In conclusion, the increased catalytic activity by descending the Group 1 originates from the decreasing interaction between cation and anion, which in turn increases the nucleophilicity of the anion, accompanied by a decreasing overall barrier of the reaction.

## Conclusion

In summary, we have presented a detailed study on the catalytic activity of crown ether coordinated alkali metal diphenyl phosphides (**1^AM^**) in the isomerisation of alkenes and alkynes, whereby we were able to show that the lightest congener **1^Li^** lacks activity. Moving down Group 1, the activity increases sequentially and significantly, which is clear to see from the respective TOFs [1 h^−1^ (**1^Na^**), 201 h^−1^ (**1^K^**), 265 h^−1^ (**1^Rb^**), and 493 h^−1^ (**1^Cs^**)]. Switching from C_6_D_6_ to the more polar solvent MeCN‐D_3_ aligned the activities of **1^K^**, **1^Rb^** and **1^Cs^** boosting K to the activity of Cs with allylbenzene **2** as a substrate accompanied by a doubling of the ***E***: ***Z*** ratios. The increase for **1^Na^** is strongest, with an elevenfold increase in TOF. Furthermore, we were able to successfully isomerise 22 activated and less activated substrates with a broad spectrum of functional groups, including aryl halides, nitriles, ethers and N‐heterocycles. However, carbonyl, thioether and alkyl halides led to decomposition of **1^Cs^**. Using terminal triple bonds led to the selective formation of allene derivatives, which then underwent further functionalisation by HP, showcasing the extra capability of the title compounds to act as rare examples of RF catalysts. Finally, through combined experimental and computational studies, we were able to construct a possible reaction mechanism for the isomerisation of allylbenzene, showing that the separation of the AM(crown) and the PPh_2_ fragment is crucial. This new study combined with our most recent research on catalytic HP chemistry^[^ ^55^, ^57^ ^]^ nicely informs us that the nucleophilicity (or basicity) of the PPh_2_ anion can be increased by several distinct strategies: i) addition of a donor ligand sequestering the cation from the anion; ii) using a heavier alkali metal as a cation; and iii) increasing the polarity of the reaction solvent. All these strategies in fact lead to a decreased interaction between the cation and the anion, increasing the nucleophilicity of the phosphide and enabling transformations that were meant to be reserved for superbasic systems but somewhat counter intuitively can be achieved with increased functional group tolerance.

## Supporting Information

Computational and Experimental details including NMR spectra of the catalytic reactions and the isolated products, SC‐XRD and refinement data are provided in the Supporting Information. Additional references are cited in the supporting information.^[^ ^66^, ^67^, ^68^, ^69^, ^70^, ^71^, ^72^, ^73^, ^74^, ^75^, ^76^, ^77^, ^78^, ^79^, ^80^, ^81^, ^82^, ^83^, ^84^, ^85^, ^86^, ^87^, ^88^, ^89^, ^90^, ^91^, ^92^, ^93^, ^94^ ^]^

## Conflict of Interests

The authors declare no conflict of interest.

## Supporting information



Supporting Information

Supporting Information

## Data Availability

Deposition number 2492230 contain the supplementary crystallographic data for this paper. These data are provided free of charge by the joint Cambridge Crystallographic Data Centre and Fachinformationszentrum Karlsruhe Access Structures service. Data that support the findings of this study are openly available in Pureportal.strath.ac.uk at https://doi.org/10.15129/7900f9e4‐2b40‐4b35‐b814‐e3506e7593fc, reference number 315734319.
