# Supplementary material for: Boosting the Nucleophilicity of the Diphenylphosphide Anion with Crown Ether Supported Heavy Alkali Metals to Facilitate Highly Efficient Catalytic Alkene Isomerisation
Source: Angew Chem Int Ed Engl. 2026 Jan 20;65(9):e23460. doi: 10.1002/anie.202523460 (PMC12930020; doi:10.1002/anie.202523460)
Supplement: Supplementary file 1 — Supporting Information [file ANIE-65-e23460-s002.docx]

**Boosting the Nucleophilicity of the Diphenylphosphide Anion with Crown Ether Supported Heavy Alkali Metals to Facilitate Highly Efficient Catalytic Alkene Isomerisation**

Felix Krämer^[a]^* Thomas Horsley M. Downie and Robert E. Mulvey^[a]^*

[a] Dr F. Krämer, Dr T. M. Horsley Downie and Prof. Dr R. E. Mulvey,

Department of Pure and Applied Chemistry, University of Strathclyde,
Glasgow G1 1XL, United Kingdom, E-mail: felix.kraemer@strath.ac.uk, r.e.mulvey@strath.ac.uk

**Supporting Information**

TOC

[Section S1 – Experimental Details 2](#_Toc215660479)

[Section S2 – NMR Spectra of **15** and **16** 5](#_Toc215660480)

[Section S3 – NMR Spectra of the Catalytic Reactions 7](#_Toc215660481)

[Section S3.1 – NMR Spectra of the **1^AM^** catalysed isomerisation of Allylbenzene **2** with (catalyst screening) 7](#_Toc215660482)

[Section S3.2 – NMR Spectra of the **1^Cs^** catalysed isomerisation of **3**-**23** 11](#_Toc215660483)

[Section S3.3 – NMR Spectra of the **1^K^** catalysed isomerisation of **3**-**17** 34](#_Toc215660484)

[Section S3.4 – NMR Spectra of the **1^Cs^** catalysed remote functionalisation of **2**, **6**, **7**, **14** and **17**-**19** 52](#_Toc215660485)

[Section S4 – DOSY NMR studies of **1^AM^** in MeCN-D_3_ 65](#_Toc215660486)

[Section S5 – Kinetic Studies 69](#_Toc215660487)

[Section S6 – Crystallographic details 75](#_Toc215660488)

[Section S7 – Computational Details 77](#_Toc215660489)

[References 79](#_Toc215660490)

# Section S1 – Experimental Details

**General Experimental Details:** All synthetic procedures were carried out under a dry nitrogen atmosphere (N_2_) using standard Schlenk techniques or in a glove box under an argon atmosphere (Ar). Before use, the glassware was pre-dried in an oven at 150 °C and then heated with a heat gun under vacuum. The solvents were dried, distilled and degassed using standard methods. C_6_D_6_ was dried over potassium, distilled, degassed and then stored in the glove box over activated molecular sieves (4 Å). MeCN-D_3_ was dried over CaH_2_, degassed and distilled and then stored in the glove box over activated molecular sieves (4 Å). n-Pentane was dried in a Solvent Purification System (Innovative Technology, PS-Micro), degassed and stored under inert atmosphere over activated 4 Å molecular sieves. **1^AM^** and *^t^*BuPhPH were synthesised as reported.^[1]^ **1^nBu4N^** and *^n^*Bu_2_PH were synthesised as reported.^[2]^ Ph_2_PH^[3]^ was synthesised according to literature procedures and was further purified by stirring over LiAlH_4_ and a second distillation. All other substrates were obtained from commercial sources and were condensed, degassed and stored over activated 4 Å molecular sieves under argon in a glove box. *^n^*BuLi (2.5 M, hexane), as obtained from commercial sources. ^1^H, ^13^C{^1^H} and ^31^P{^1^H} NMR spectra were recorded using an AV400 or AV500 MHz spectrometer. The chemical shifts (*δ* in ppm) in the ^1^H and ^13^C NMR spectra were referenced to the residual signals of the deuterated solvents. ^1^H and ^13^C{^1^H} chemical shifts were reported against Me_4_Si and ^31^P{^1^H} against H_3_PO_4_. Common abbreviations were used to describe the signal multiplications: s (singlet), d (doublet), t (triplet), q (quartet), dd (doublet of a doublet), m (multiplet) and br (broad).

**Synthesis of Allyl-PPh_2_ (14)**: Under a nitrogen atmosphere Ph_2_PH (3.21 g, 3.00 mL, 17.24 mmol, 1 eq.) was dissolved in *n*-pentane (20 mL) and cooled to 0 °C with an ice bath. Subsequently *^n^*BuLi (2.5 M, 7.60 mL, 18.96 mmol, 1.1 eq.) was added dropwise via syringe whereupon a bright yellow solid formed. After the reaction mixture was stirred at RT for 30 min the suspension was cooled to 0 °C again and Allylbromide (2.29 g, 1.64 mL, 18.96 mmol, 1.1 eq.) was added dropwise. The colourless suspension was stirred overnight at RT, cannula filtered and the remaining solids were extracted with 10 ml *n*-pentane. The solvent was removed under reduced pressure yielding 3.4 g (87 %) of 14 as a colourless oil. The NMR data fits well with the reported data.^[4]^

**Synthesis of Allyl-PPh*^t^*Bu (15)**: Under a nitrogen atmosphere Ph*^t^*BuPH (1.85 g, 2.00 mL, 11.13 mmol, 1 eq.) was dissolved in *n*-pentane (20 mL) and cooled to 0 °C with an ice bath. Subsequently *^n^*BuLi (2.5 M, 4.90 mL, 12.24 mmol, 1.1 eq.) was added dropwise via syringe whereupon a colourless solid formed. After the reaction mixture was stirred at RT for 2 h the suspension was cooled to 0 °C again and Allylbromide (1.48 g, 1.06 mL, 12.24 mmol, 1.1 eq.) was added dropwise. The colourless suspension was stirred for 4 hours at RT and syringe filtered. The solvent was removed under reduced pressure to 50 % whereupon a colourless solid forms which was identified as (Ph*^t^*BuP)_2_. The mixture was stored at -30 °C for three days. The solution was removed, the colourless crystals were discarded and the solvent was removed under reduced pressure. The remaining residue was distilled (trap to trap) in vacuum yielding **15** as colourless oil (546 mg, 24 %) containing 12 % Ph*^t^*BuPH. ^1^H NMR (400 MHz, 300 K, C_6_D_6_, ppm): *δ* = 7.49 - 7.42 (m, H_Ar_ 2H), 7.19 - 7.06 (m, H_Ar_, 3H), 5.92 - 5.76 (m, CH, 1H), 5.12 - 5.01 and 4.95 - 4.85 (m, CH_2_ (Allyl), 2H), 2.70 - 2.34 (m, CH_2_, 2H), 0.93 (d, ^3^*J_PH_* = 11.6 Hz, H*_t_*_Bu_, 9H). ^31^P NMR (121 MHz, 300 K, C_6_D_6_, ppm): *δ* = 2.9 (s).

**Synthesis of Allyl-P*^n^*Bu_2_ (16)**: Under a nitrogen atmosphere *^n^*Bu_2_PH (2.36 g, 3.00 mL, 16.17 mmol, 1 eq.) was dissolved in *n*-pentane (20 mL) and cooled to 0 °C with an ice bath. Subsequently *^n^*BuLi (2.5 M, 7.11 mL, 17.78 mmol, 1.1 eq.) was added dropwise via syringe. After the reaction mixture was stirred at RT for 2 h the colourless suspension was cooled to 0 °C again and Allylbromide (2.15 g, 1.54 mL, 17.78 mmol, 1.1 eq.) was added dropwise. The colourless suspension was stirred overnight at RT, cannula filtered and the solvent was removed under reduced pressure. The remaining residue was distilled (trap to trap) in vacuum yielding **16** as colourless oil (1.46 g, 49 %). ^1^H NMR (400 MHz, 300 K, C_6_D_6_, ppm): *δ* = 5.86 - 5.70 (m, CH, 1H), 5.04 - 4.90 (m, CH_2_ (Allyl), 2H), 2.13 (m, CH_2_, 2H), 1.48 - 1.18 (m, CH_2_ (*^n^*Bu), 12H) , 0.91 – 0.83 (m, CH_3_ (*^n^*Bu), 6H). ^31^P NMR (121 MHz, 300 K, C_6_D_6_, ppm): *δ* = -32.8 (s).

**General procedure for reaction monitoring of catalytic reactions**: The experiments were made on a 1 mmol of Allylbenzene **2**. In a Young NMR tube 0.5 mL C_6_D_6_ containing C_6_Me_6_ (100 μmol/mL) or 0.5 mL MeCN-D_3_ (with 21 μL Toluene) and the substrate were combined, and the NMR spectrometer was tuned. Then the catalyst (10 μmol) was placed in the NMR tube so that nothing can dissolve. Directly in front of the spectrometer the probe was shaken and put into the spectrometer. NMR spectra were recorded every minute, every 30 min for Na respectively.


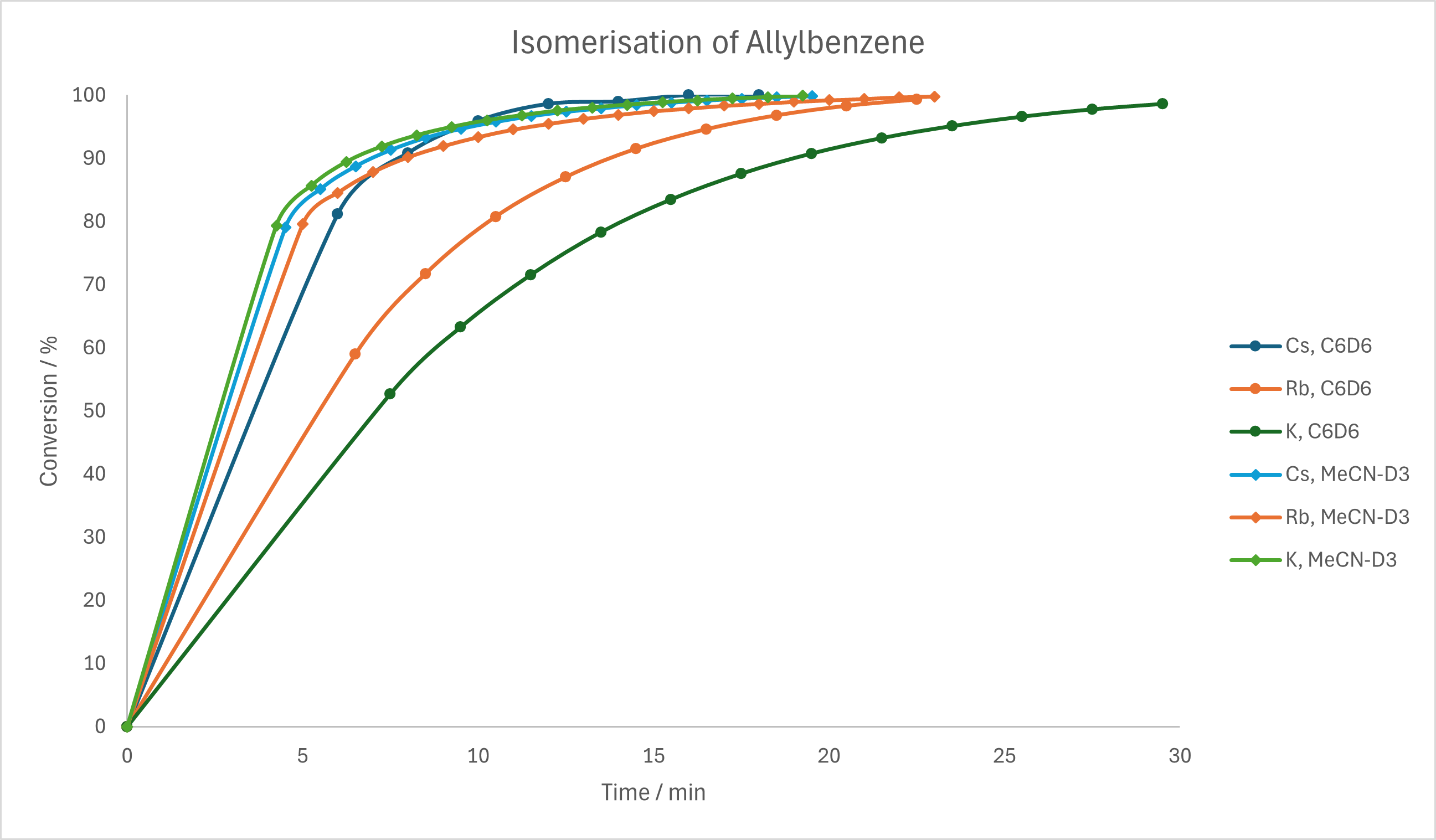

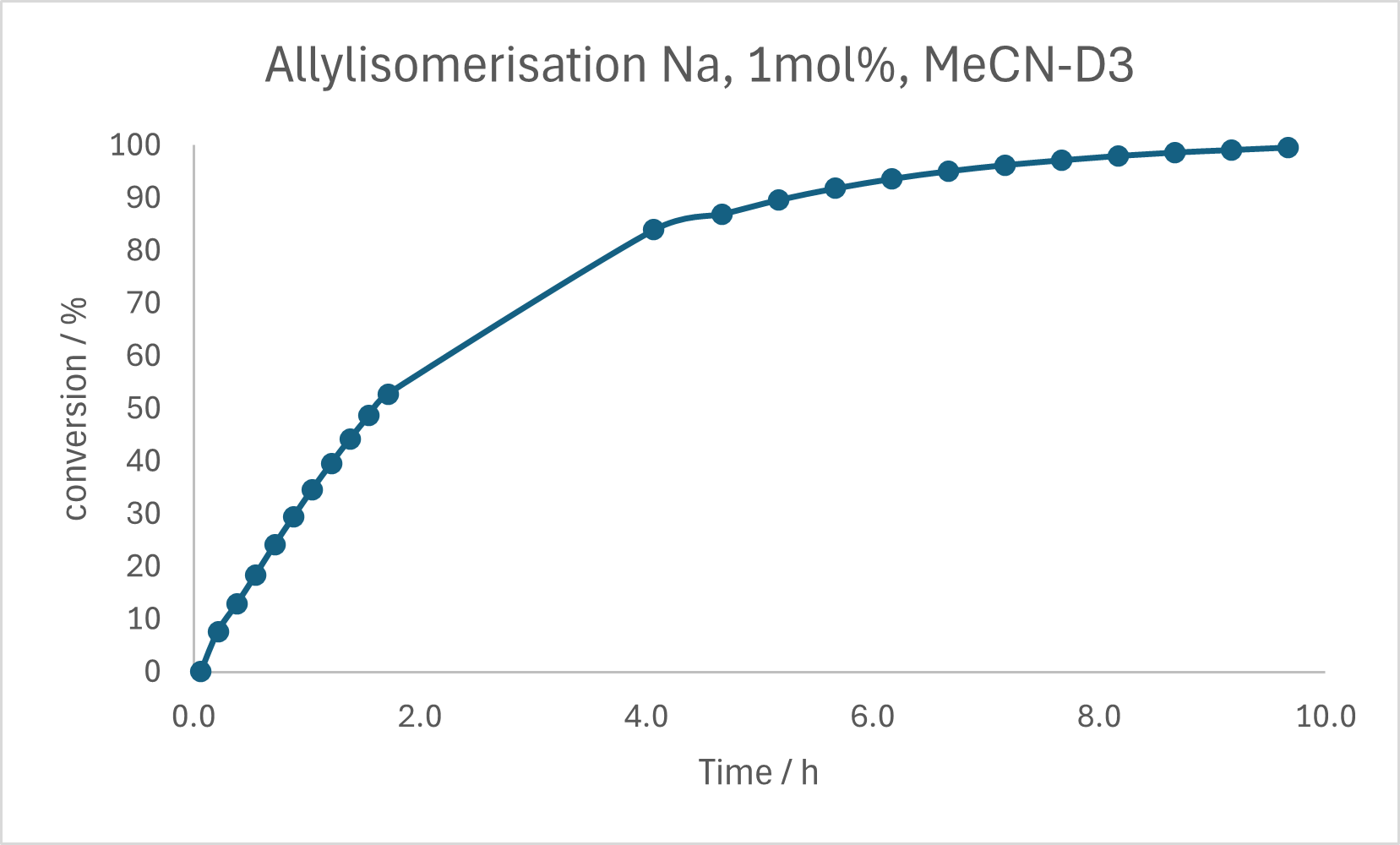


Figure S1. Plotted conversion vs time for the **1^AM^** catalysed isomerisation of **2** in C_6_D_6_ and MeCN-D_3_ at 300K.

**General procedure for catalytic reactions in C_6_D_6_**: The catalytic experiments were made on a 500 μmol scale for the substrates. In a Young NMR tube 0.5 mL C_6_D_6_ containing C_6_Me_6_ (100 μmol/mL) and the substrate were combined, and NMR spectra were recorded. Subsequently the catalyst (25 - 100 μmol) was added as a solid and the NMR tube was shaken until the catalyst was fully dissolved. Details about reaction times and temperatures are listed in Table S1. Conditions: [a] 1 mol%, RT, 30 min; [a*]: 8 h instead of 30 min; [a**]: 1 h instead of 30 min; [b] 5 mol%, R, 30 min; [c] 5 mol%, RT 4 h; [d] 5 mol%, 60 °C, 18 h; [d*]: 3 h instead of 18 h; [e] 5 mol%, 80 °C, 40 h; [e*]: 4 d instead of 40 h; [e**] 19 h instead of 40 h; [e***] 24 h instead of 40 h; [f] MeCN-d_3_ 5 mol%, 80 °C, 19 h.

Table S1. Reaction conditions, times and yields of the **1^Cs^** catalysed isomerisation of substrates **2**-**23**. (s.o.: signal overlap)

|  |  |  |  |  | **1^Cs^** | | **1^K^** | |  |
| --- | --- | --- | --- | --- | --- | --- | --- | --- | --- |
| **Compound** | **No** | **Mol%** | **T / °C** | **Time** | **yield** | **E:Z** | **yield** | **E:Z** | **conditions** |
| Allylbenzene | **2** | **1** | RT | 12 min | 99 | 11:1 | 99 | 12:1 | a |
| Allylanisole | **3** | **1** | RT | 8 h | 85 | 6:1 | 18 | 7:1 | a* |
| p-F-Allylbenzene | **4** | **5** | RT | 30 min | 99 | s.o. | 78 | 9:1 | b |
| o-Br-Allylbenzene | **5** | **5** | RT | 30 min | 99 | 12:1 | 99 | 12:1 | b |
| Allylnaphtalene | **6** | **1** | RT | 30 min | 99 | 9:1 | 8 | - | a |
| Allylcarbazole | **7** | **5** | RT | 3 h | 90 | 5:1 | 68 | 3:1 | c |
| Allyldimethylpyrazole | **8** | **5** | RT | 4 h | 88 | s.o. | 40 | 7:1 | c |
| Allyl-CN | **9** | **1** | RT | 30 min | 99 | 3:2 | 99 | 5:4 | a |
| Allylethylether | **10** | **5** | 60 | 18 h | 99 | - | 62 | - | d |
| 1,4-Cyclohexadiene | **11** | **5** | 60 | 18 h | 82 | - | 75 | - | d |
| Allylamine | **12** | **5** | 80 | 40 h | 88 | 1:1 | 21 | - | e |
| Allyldimethylamine | **13** | **5** | 80 | 4 d | 5 | - | 7 | - | e* |
| Allyl-PPh2 | **14** | **5** | RT | 1 h | 99 | 11:1 | 99 | 16:1 | a** |
| Allyl-PtBuPh | **15** | **5** | RT | 45 h | 99 | 17:1 | 99 | - | a** |
| Allyl-PnBu2 | **16** | **5** | 60 | 3 h | 99 | 8:1 | 76 | 13:1 | d* |
| 1,1-Dimethyl-3-phenylpropene | **17** | **5** | 80 | 24 h | 57 | - | 32 | - | e*** |
| Allyl-TMS | **18** | **5** | 80 (MeCN) | 19 h | 70 | 1:1 | 71 | 13:1 | f |
| 3-Methylcyclohexane | **19** | **5** | 80 (MeCN) | 19 h | 0 | - | - | - | f |
| 1-Hexene | **20** | **5** | 80 (MeCN) | 19 h | 0 | - | - | - | f |
| Ph-CH2-CC-H | **21** | **5** | RT | 30 min | 88 | - | - | - | b |
| Ph-CH2-CH2-CC-H | **22** | **5** | RT | 3 h | 99 | - | - | - | c |
| Me2N-CH2-CC-H | **23** | **5** | RT | 30 min | 90 | - | - | - | b |
| 1-Hexyne | **24** | **5** | RT | 30 min | 99 | - | - | - | a |
| 3-Hexyne | **25** | **5** | 80 | 19 h | 52 | - | - | - | e** |

**General procedure for catalytic remote functionalisation reactions in C_6_D_6_**: The catalytic experiments were made on a 500 μmol scale for the substrates and phosphines. In a Young NMR tube 0.5 mL C_6_D_6_ containing C_6_Me_6_ (100 μmol/mL) and the substrate were combined, and NMR spectra were recorded. Subsequently the catalyst (100 μmol) was added as a solid and the NMR tube was shaken until the catalyst was fully dissolved. The reaction mixtures were stored at RT for 2 h for the allyl derivatives **2**, **6**, **7** and **14** and. Subsequently Ph_2_PH or *^n^*Bu_2_PH was added and the rection mixtures were heated to 90 °C for 4 h. For the Alkyne substrates **17**-**19** the isomerisation was given 3 h at RT and then Ph_2_PH was added. After a maximum of 2 h the reactions were complete.

# Section S2 – NMR Spectra of **15** and **16**

Figure S2. ^1^H NMR spectrum of **15** in C_6_D_6_ at 300K. With * marked signals belong remaining Ph^t^BuPH.

Figure S3. ^31^P{^1^H} NMR spectrum of **15** in C_6_D_6_ at 300K. Signal at -5.68 belong remaining Ph^t^BuPH.

Figure S4. ^1^H NMR spectrum of **16** in C_6_D_6_ at 300K.

Figure S5. ^31^P{^1^H} NMR spectrum of **16** in C_6_D_6_ at 300K. Signal at -69.4 belong remaining ^n^Bu_2_PH, Signals at -42.5 and -32.1 belong to unknown impurities.

# Section S3 – NMR Spectra of the Catalytic Reactions

## Section S3.1 – NMR Spectra of the **1^AM^** catalysed isomerisation of Allylbenzene **2** with (catalyst screening)

Figure S6. ^1^H NMR Spectra (C_6_D_6_, 300K) of the **1^AM^** catalysed isomerisation of Allylbenzene **2**.

Figure S7. ^1^H NMR Spectra (MeCN-D_3_, 300K) of the **1^AM^** catalysed isomerisation of Allylbenzene **2**.

For the determination of the E:Z ratios the reactions in MeCN the solvent was removed after the reaction was finished and the residue was dissolved in C_6_D_6_.

Figure S8. ^1^H NMR Spectra (C_6_D_6_, 300K) of the **1^Na^** catalysed isomerisation of Allylbenzene **2** carried out in MeCN.

Figure S9. ^1^H NMR Spectra (C_6_D_6_, 300K) of the **1^K^** catalysed isomerisation of Allylbenzene **2** carried out in MeCN.

Figure S10. ^1^H NMR Spectra (C_6_D_6_, 300K) of the **1^Rb^** catalysed isomerisation of Allylbenzene **2** carried out in MeCN.

Figure S11. ^1^H NMR Spectra (C_6_D_6_, 300K) of the **1^Cs^** catalysed isomerisation of Allylbenzene **2** carried out in MeCN.

Figure S12. ^1^H NMR Spectra (C_6_D_6_, 300K) of the **1^nBu4N^** catalysed isomerisation of Allylbenzene **2**. No conversion after 24 h at RT.

## Section S3.2 – NMR Spectra of the **1^Cs^** catalysed isomerisation of **3**-**23**

Figure S13. ^1^H NMR spectrum of **1^Cs^** catalysed isomerisation of **3** in C_6_D_6_ at 300K. * Signals belong to substrate **3**.

Figure S14.Stacked ^1^H NMR spectrum of **3** before reaction (Top) and **1^Cs^** catalysed isomerisation of **3** in C_6_D_6_ at 300K (bottom).

Figure S15. ^1^H NMR spectrum of **1^Cs^** catalysed isomerisation of **4** in C_6_D_6_ at 300K. * Signals belong to substrate **4**.

Figure S16. Stacked ^1^H NMR spectrum of **4** before reaction (Top) and **1^Cs^** catalysed isomerisation of **4** in C_6_D_6_ at 300K (bottom).

Figure S17. ^1^H NMR spectrum of **1^Cs^** catalysed isomerisation of **5** in C_6_D_6_ at 300K. * Signals belong to substrate **5**.

Figure S18. Stacked ^1^H NMR spectrum of **5** before reaction (Top) and **1^Cs^** catalysed isomerisation of **5** in C_6_D_6_ at 300K (bottom).

Figure S19. ^1^H NMR spectrum of **1^Cs^** catalysed isomerisation of **6** in C_6_D_6_ at 300K.

Figure S20. Stacked ^1^H NMR spectrum of **6** before reaction (Top) and **1^Cs^** catalysed isomerisation of **6** in C_6_D_6_ at 300K (bottom).

Figure S21. ^1^H NMR spectrum of **1^Cs^** catalysed isomerisation of **7** in C_6_D_6_ at 300K. * Signals belong to substrate **7**.

Figure S22. Stacked ^1^H NMR spectrum of **6** before reaction (Top) and **1^Cs^** catalysed isomerisation of **6** in C_6_D_6_ at 300K (bottom).

Figure S23. ^1^H NMR spectrum of **1^Cs^** catalysed isomerisation of **8** in C_6_D_6_ at 300K. * Signals belong to substrate **8**.

Figure S24. Stacked ^1^H NMR spectrum of **8** before reaction (Top) and **1^Cs^** catalysed isomerisation of **8** in C_6_D_6_ at 300K (bottom).

Figure S25. ^1^H NMR spectrum of **1^Cs^** catalysed isomerisation of **9** in C_6_D_6_ at 300K.

Figure S26. Stacked ^1^H NMR spectrum of **9** before reaction (Top) and **1^Cs^** catalysed isomerisation of **9** in C_6_D_6_ at 300K (bottom).

Figure S27. ^1^H NMR spectrum of **1^Cs^** catalysed isomerisation of **10** in C_6_D_6_ at 300K. * Signals belong to substrate **10**.

Figure S28. Stacked ^1^H NMR spectrum of **10** before reaction (Top) and **1^Cs^** catalysed isomerisation of **10** in C_6_D_6_ at 300K (bottom).

Figure S29. ^1^H NMR spectrum of **1^Cs^** catalysed isomerisation of **11** in C_6_D_6_ at 300K. * Signals belong to substrate **11**.

Figure S30. Stacked ^1^H NMR spectrum of **11** before reaction (Top) and **1^Cs^** catalysed isomerisation of **11** in C_6_D_6_ at 300K (bottom).

Figure S31. ^1^H NMR spectrum of **1^Cs^** catalysed isomerisation of **12** in C_6_D_6_ at 300K. * Signals belong to substrate **12**.

Figure S32. Stacked ^1^H NMR spectrum of **12** before reaction (Top) and **1^Cs^** catalysed isomerisation of **12** in C_6_D_6_ at 300K (bottom).

Figure S33. Stacked ^1^H NMR spectrum of **13** before reaction (Top) and **1^Cs^** catalysed isomerisation of **13** (5 % conversion) in C_6_D_6_ at 300K (bottom).

Figure S34. ^1^H NMR spectrum of **1^Cs^** catalysed isomerisation of **14** in C_6_D_6_ at 300K.

Figure S35. Stacked ^1^H NMR spectrum of **14** before reaction (Top) and **1^Cs^** catalysed isomerisation of **14** in C_6_D_6_ at 300K (bottom).

Figure S36. ^31^P{^1^H} NMR spectrum of **1^Cs^** catalysed isomerisation of **14** in C_6_D_6_ at 300K. Signal at 16 ppm belongs to starting material. Signal at -32 belongs to unknown impurity.

Figure S37. ^1^H NMR spectrum of **1^Cs^** catalysed isomerisation of **15** in C_6_D_6_ at 300K. With * marked signals belong to Ph^t^BuPH.

Figure S38. Stacked ^1^H NMR spectrum of **15** before reaction (Top) and **1^Cs^** catalysed isomerisation of **15** in C_6_D_6_ at 300K (bottom).

Figure S39. ^31^P NMR spectrum of **1^Cs^** catalysed isomerisation of **15** in C_6_D_6_ at 300K. Signal at 2.5 ppm belongs to E product, signal at 1.5 belongs to Z product. Signal at -6 belongs to Ph^t^BuPH. Signal at -17 belongs to unknown impurity.

Figure S40. ^1^H NMR spectrum of **1^Cs^** catalysed isomerisation of **16** in C_6_D_6_ at 300K.

Figure S41. Stacked ^1^H NMR spectrum of **16** before reaction (Top) and **1^Cs^** catalysed isomerisation of **16** in C_6_D_6_ at 300K (bottom).

Figure S42. ^31^P{^1^H} NMR spectrum of **1^Cs^** catalysed isomerisation of **16** in C_6_D_6_ at 300K. Signal at 31 ppm belongs to E product. Signals -70 and -42 belong to impurities from starting material. Signal at -53 ppm belongs to unknown impurity.

Figure S43. ^1^H NMR spectrum of **1^Cs^** catalysed isomerisation of **17** in C_6_D_6_ at 300K.

Figure S44. Stacked ^1^H NMR spectrum of **17** before reaction (Top) and **1^Cs^** catalysed isomerisation of **17** in C_6_D_6_ at 300K (bottom).

Figure S45. ^1^H NMR spectrum of **1^Cs^** catalysed isomerisation of **18** in MeCN-D_3_ at 300K.

Figure S46. Stacked ^1^H NMR spectrum of **18** before reaction (Top) and **1^Cs^** catalysed isomerisation of **19** in MeCN-D_3_ at 300K after 1 h at 80 °C (bottom).

Figure S47. Stacked ^1^H NMR spectrum of **18** before reaction (Top) and **1^Cs^** catalysed isomerisation of **18** in MeCN-D_3_ at 300K (bottom).

Figure S48. Stacked ^1^H NMR spectrum of 1-hexene **20** before reaction (Top) and **1^Cs^** catalysed isomerisation of **20** after 24 h at 80 °C in MeCN-D_3_ at 300K (bottom).

Figure S49. ^1^H NMR spectrum of **1^Cs^** catalysed isomerisation of **21** in C_6_D_6_ at 300K. * belongs to Me of the internal alkyne.

Figure S50. Stacked ^1^H NMR spectrum of **21** before reaction (Top) and **1^Cs^** catalysed isomerisation of **21** in C_6_D_6_ at 300K (bottom).

Figure S51. ^1^H NMR spectrum of **1^Cs^** catalysed isomerisation of **22** in C_6_D_6_ at 300K.

Figure S52. Stacked ^1^H NMR spectrum of **22** before reaction (Top) and **1^Cs^** catalysed isomerisation of **22** in C_6_D_6_ at 300K (bottom).

Figure S53. ^1^H NMR spectrum of **1^Cs^** catalysed isomerisation of **23** in C_6_D_6_ at 300K. * belongs to internal alkyne.

Figure S54. Stacked ^1^H NMR spectrum of **23** before reaction (Top) and **1^Cs^** catalysed isomerisation of **23** in C_6_D_6_ at 300K (bottom).

Figure S55. ^1^H NMR spectrum of **1^Cs^** catalysed isomerisation of **24** in C_6_D_6_ at 300K.

Figure S56. Stacked ^1^H NMR spectrum of **24** before reaction (Top) and **1^Cs^** catalysed isomerisation of **24** in C_6_D_6_ at 300K (bottom).

Figure S57. ^1^H NMR spectrum of **1^Cs^** catalysed isomerisation of **25** in C_6_D_6_ at 300K.

Figure 58. Stacked ^1^H NMR spectrum of **25** before reaction (Top) and **1^Cs^** catalysed isomerisation of **25** in C_6_D_6_ at 300K (bottom).

## Section S3.3 – NMR Spectra of the **1^K^** catalysed isomerisation of **3**-**17**

Figure S59. ^1^H NMR spectrum of **1^K^** catalysed isomerisation of **3** in C_6_D_6_ at 300K. * Signals belong to substrate **3**.

Figure S60.Stacked ^1^H NMR spectrum of **3** before reaction (Top) and **1^K^** catalysed isomerisation of **3** in C_6_D_6_ at 300K (bottom).

Figure S61. ^1^H NMR spectrum of **1^K^** catalysed isomerisation of **4** in C_6_D_6_ at 300K. * Signals belong to substrate **4**.

Figure S62. Stacked ^1^H NMR spectrum of **4** before reaction (Top) and **1^K^** catalysed isomerisation of **4** in C_6_D_6_ at 300K (bottom).

Figure S63. ^1^H NMR spectrum of **1^K^** catalysed isomerisation of **5** in C_6_D_6_ at 300K. * Signals belong to substrate **5**.

Figure S64. Stacked ^1^H NMR spectrum of **5** before reaction (Top) and **1^K^** catalysed isomerisation of **5** in C_6_D_6_ at 300K (bottom).

Figure S65. ^1^H NMR spectrum of **1^K^** catalysed isomerisation of **6** in C_6_D_6_ at 300K.

Figure S66. Stacked ^1^H NMR spectrum of **6** before reaction (Top) and **1^K^** catalysed isomerisation of **6** in C_6_D_6_ at 300K (bottom).

Figure S67. ^1^H NMR spectrum of **1^K^** catalysed isomerisation of **7** in C_6_D_6_ at 300K. * Signals belong to substrate **7**.

Figure S68. Stacked ^1^H NMR spectrum of **6** before reaction (Top) and **1^K^** catalysed isomerisation of **6** in C_6_D_6_ at 300K (bottom).

Figure S69. ^1^H NMR spectrum of **1^K^** catalysed isomerisation of **8** in C_6_D_6_ at 300K. * Signals belong to substrate **8**.

Figure S70. Stacked ^1^H NMR spectrum of **8** before reaction (Top) and **1^K^** catalysed isomerisation of **8** in C_6_D_6_ at 300K (bottom).

Figure S71. ^1^H NMR spectrum of **1^K^** catalysed isomerisation of **9** in C_6_D_6_ at 300K.

Figure S72. Stacked ^1^H NMR spectrum of **9** before reaction (Top) and **1^K^** catalysed isomerisation of **9** in C_6_D_6_ at 300K (bottom).

Figure S73. ^1^H NMR spectrum of **1^K^** catalysed isomerisation of **10** in C_6_D_6_ at 300K. * Signals belong to substrate **10**.

Figure S74. Stacked ^1^H NMR spectrum of **10** before reaction (Top) and **1^K^** catalysed isomerisation of **10** in C_6_D_6_ at 300K (bottom).

Figure S75. ^1^H NMR spectrum of **1^K^** catalysed isomerisation of **11** in C_6_D_6_ at 300K. * Signals belong to substrate **11**.

Figure S76. Stacked ^1^H NMR spectrum of **11** before reaction (Top) and **1^K^** catalysed isomerisation of **11** in C_6_D_6_ at 300K (bottom).

Figure S77. ^1^H NMR spectrum of **1^K^** catalysed isomerisation of **12** in C_6_D_6_ at 300K. * Signals belong to substrate **12**.

Figure S78. Stacked ^1^H NMR spectrum of **12** before reaction (Top) and **1^K^** catalysed isomerisation of **12** in C_6_D_6_ at 300K (bottom).

Figure S79. ^1^H NMR spectrum of **1^K^** catalysed isomerisation of **13** in C_6_D_6_ at 300K. * Signals belong to substrate **12**.

Figure S80. Stacked ^1^H NMR spectrum of **13** before reaction (Top) and **1^K^** catalysed isomerisation of **13** (5 % conversion) in C_6_D_6_ at 300K (bottom).

Figure S81. ^1^H NMR spectrum of **1^K^** catalysed isomerisation of **14** in C_6_D_6_ at 300K.

Figure S82. Stacked ^1^H NMR spectrum of **14** before reaction (Top) and **1^K^** catalysed isomerisation of **14** in C_6_D_6_ at 300K (bottom).

Figure S83. ^31^P{^1^H} NMR spectrum of **1^K^** catalysed isomerisation of **14** in C_6_D_6_ at 300K. Signal at 16 ppm belongs to starting material. Signal at -32 belongs to unknown impurity.

Figure S84. ^1^H NMR spectrum of **1^K^** catalysed isomerisation of **15** in C_6_D_6_ at 300K. With * marked signals belong to Ph^t^BuPH.

Figure S85. Stacked ^1^H NMR spectrum of **15** before reaction (Top) and **1^K^** catalysed isomerisation of **15** in C_6_D_6_ at 300K (bottom).

Figure S86. ^31^P NMR spectrum of **1^K^** catalysed isomerisation of **15** in C_6_D_6_ at 300K. Signal at 2.5 ppm belongs to E product. Signal at -5.5 belongs to Ph^t^BuPH. Signal at -17 belongs to unknown impurity.

Figure S87. ^1^H NMR spectrum of **1^K^** catalysed isomerisation of **16** in C_6_D_6_ at 300K.

Figure S88. Stacked ^1^H NMR spectrum of **16** before reaction (Top) and **1^K^** catalysed isomerisation of **16** in C_6_D_6_ at 300K (bottom).

Figure S89. ^31^P{^1^H} NMR spectrum of **1^K^** catalysed isomerisation of **16** in C_6_D_6_ at 300K. Signal at 31 ppm belongs to E product. Signals -70 and -42 belong to impurities from starting material. Signal at -53 ppm belongs to unknown impurity.

Figure S90. ^1^H NMR spectrum of **1^K^** catalysed isomerisation of **17** in C_6_D_6_ at 300K.

Figure S91. Stacked ^1^H NMR spectrum of **17** before reaction (Top) and **1^K^** catalysed isomerisation of **17** in C_6_D_6_ at 300K (bottom).

Figure S92. ^1^H NMR spectrum of **1^K^** catalysed isomerisation of **18** in MeCN-D_3_ at 300K.

Figure S93. Stacked ^1^H NMR spectrum of **18** before reaction (Top) and **1^K^** catalysed isomerisation of **17** in MeCN-D_3_ at 300K (bottom).

## Section S3.4 – NMR Spectra of the **1^Cs^** catalysed remote functionalisation of **2**, **6**, **7**, **14** and **17**-**19**

Figure S94. ^1^H NMR spectrum of **1^Cs^** catalysed remote functionalisation of **2** with Ph_2_PH in C_6_D_6_ at 300K.

Figure S95. Stacked ^1^H NMR spectrum of isomerised **2** before HP reaction (Top) and **1^Cs^** catalysed hydrophosphination of isomerised **2** with Ph_2_PH in C_6_D_6_ at 300K (bottom).

Figure S96. ^31^P{^1^H} NMR spectrum of RF of **2** with Ph_2_PH in C_6_D_6_ at 300K.

Figure S97. ^1^H NMR spectrum of **1^Cs^** catalysed remote functionalisation of **2** with ^n^Bu_2_PH in C_6_D_6_ at 300K.

Figure S98. Stacked ^1^H NMR spectrum of isomerised **2** before HP reaction (Top) and **1^Cs^** catalysed hydrophosphination of isomerised **2** with ^n^Bu_2_PH in C_6_D_6_ at 300K (bottom).

Figure S99. ^31^P{^1^H} NMR spectrum of RF of **2** with ^n^Bu_2_PH in C_6_D_6_ at 300K. Signal at -70 belongs to ^n^Bu_2_PH.

Figure S100. ^1^H NMR spectrum of **1^Cs^** catalysed remote functionalisation of **6** with Ph_2_PH in C_6_D_6_ at 300K.

Figure S101. Stacked ^1^H NMR spectrum of isomerised **3** before HP reaction (Top) and **1^Cs^** catalysed hydrophosphination of isomerised **3** with Ph_2_PH in C_6_D_6_ at 300K (bottom).

Figure S102. ^31^P{^1^H} NMR spectrum of RF of **3** with Ph_2_PH in C_6_D_6_ at 300K.

Figure S103. ^1^H NMR spectrum of **1^Cs^** catalysed remote functionalisation of **6** with ^n^Bu_2_PH in C_6_D_6_ at 300K.

Figure S104. Stacked ^1^H NMR spectrum of isomerised **6** before HP reaction (Top) and **1^Cs^** catalysed hydrophosphination of isomerised **6** with ^n^Bu_2_PH in C_6_D_6_ at 300K (bottom).

Figure S105. ^31^P{^1^H} NMR spectrum of RF of **6** with ^n^Bu_2_PH in C_6_D_6_ at 300K. Signal at -70 belongs to ^n^Bu_2_PH.

Figure S106. Stacked ^1^H NMR spectrum of isomerised **7** before HP reaction (Top) and **1^Cs^** catalysed hydrophosphination of isomerised **7** with Ph_2_PH in C_6_D_6_ at 300K, no reaction (bottom).

Figure S107. ^31^P{^1^H} NMR spectrum of RF of **7** with Ph_2_PH in C_6_D_6_ at 300K. no reaction.

Figure S108. ^1^H NMR spectrum of **1^Cs^** catalysed remote functionalisation of **14** with Ph_2_PH in C_6_D_6_ at 300K.

Figure S109. Stacked ^1^H NMR spectrum of isomerised **14** before HP reaction (Top) and **1^Cs^** catalysed hydrophosphination of isomerised **14** with Ph_2_PH in C_6_D_6_ at 300K (bottom).

Figure S110. ^31^P{^1^H} NMR spectrum of RF of **14** with Ph_2_PH in C_6_D_6_ at 300K. Smaller signal belong to impurities of starting materials and Ph_2_PH (-40 ppm).

Figure S111. ^1^H NMR spectrum of **1^Cs^** catalysed remote functionalisation of **21** with Ph_2_PH in C_6_D_6_ at 300K.

Figure S112. Stacked ^1^H NMR spectrum of isomerised **21** before HP reaction (Top) and **1^Cs^** catalysed hydrophosphination of isomerised **21** with Ph_2_PH in C_6_D_6_ at 300K (bottom).

Figure S113. ^31^P{^1^H} NMR spectrum of RF of **21** with Ph_2_PH in C_6_D_6_ at 300K. Z product (-13 ppm), E product (8.7 ppm), α product (-2.4 ppm), Ph_2_PH (-40 ppm)

Figure S114. ^1^H NMR spectrum of **1^Cs^** catalysed remote functionalisation of **22** with Ph_2_PH in C_6_D_6_ at 300K. No product distribution could be determined.

Figure S115. Stacked ^1^H NMR spectrum of isomerised **22** before HP reaction (Top) and **1^Cs^** catalysed hydrophosphination of isomerised **22** with Ph_2_PH in C_6_D_6_ at 300K (bottom).

Figure S116. ^31^P{^1^H} NMR spectrum of RF of **22** with Ph_2_PH in C_6_D_6_ at 300K. No product distribution could be determined.

Figure S117. ^1^H NMR spectrum of **1^Cs^** catalysed remote functionalisation of **23** with Ph_2_PH in C_6_D_6_ at 300K. * belong to the E product. + belongs to the Z and α product and could not be separated.

Figure S118. Stacked ^1^H NMR spectrum of isomerised **23** before HP reaction (Top) and **1^Cs^** catalysed hydrophosphination of isomerised **23** with Ph_2_PH in C_6_D_6_ at 300K (bottom).

Figure S119. ^31^P{^1^H} NMR spectrum of RF of **23** with Ph_2_PH in C_6_D_6_ at 300K. Z product (-14.2 ppm), E product (-13.7 ppm), α product (16.8 ppm), Ph_2_PH (-40 ppm).

# Section S4 – DOSY NMR studies of **1^AM^** in MeCN-D_3_

2D ^1^H Diffusion-Ordered Spectroscopy (DOSY) spectra were recorded on a Bruker AV400 spectrometer operating at 400.1 MHz for ^1^H and measured at 300 K. A 30 mM solution of the title compounds in 0.5 mL acetonitrile-d_3_ and 0.01 mL toluene as an internal standard. The diffusion coefficients D_c_ and D_a_ were obtained by analysing the recorded data with the Dynamic Centre program package from Bruker and listed in Table S2. An estimate of the molecular weight (MW) of the species in solution was obtained *via* comparison of the diffusion coefficients of the compounds and internal standard toluene to external calibration curves (ECCs) with normalised diffusion coefficients.^[5-7]^ The ECCs for molecules which diffuse like compact spheres (CS), dissipated spheres and ellipsoids (DSE), extended discs (ED) and a merge of all three were utilised. For species with multiple ^1^H signals, the average diffusion coefficient was taken. The accuracy of this estimation is in the range of MW_dif_ ± 9 %.^1^ In addition, the corresponding hydrodynamic radii r_H_ were calculated according to the Stokes-Einstein equation (Eq. 1) with a specific viscosity for C_6_D_6_ of *η* = 0.4047·10^-3^ kg m^2^ s^-2^. The degree of ion pairing can be determined using the quotient of the cationic (Dc) and anionic (Da) diffusion coefficients. A value of Dc/Da = 1 indicates a bond between anionic and cationic fragments, a deviation from the unit value is an indication of a certain degree of solvent-separated ion pairs (SSIPs).^[8-11]^

$r_{H}=\frac{k_{B}T}{6\pi\eta D}$ Eq. 1

Table S2. Measured DOSY-NMR data.

|  | **r_Ha_ / Å** | **r_Hc_ / Å** | Da / ^-10^m^2^/s | Dc / ^-10^m^2^/s | D_Tol_ / ^-10^m^2^/s | D_c_/D_a_ |  | D_c_/D_a_ (C_6_D_6_)^[1]^ |
| --- | --- | --- | --- | --- | --- | --- | --- | --- |
| **1Na** | 3.74 | 3.35 | 1.62E-09 | 1.45E-09 | 2.91E-09 | 0.90 |  | 1.00 |
| **1K** | 4.14 | 3.31 | 1.64E-09 | 1.31E-09 | 2.91E-09 | 0.80 |  | 0.97 |
| **1Rb** | 4.18 | 3.29 | 1.65E-09 | 1.30E-09 | 2.91E-09 | 0.79 |  | 1.01 |
| **1Cs** | 4.28 | 3.29 | 1.65E-09 | 1.27E-09 | 2.91E-09 | 0.77 |  | 0.99 |

Table S3. Molecular weights of AM(crown) and PPh_2_ in solution calculated by Stalkes method.

|  | **Cation** | | | **Anion** | | |
| --- | --- | --- | --- | --- | --- | --- |
|  | **MW_exp_ / g/mol** | **MW_calc_ / g/mol** | **MW_diff_** | **MW_exp_ / g/mol** | **MW_calc_ / g/mol** | **MW_diff_** |
| **1Na** | 290 | 243 | -16% | 241 | 185 | -23% |
| **1K** | 345 | 303 | -12% | 236 | 185 | -22% |
| **1Rb** | 349 | 350 | 0% | 233 | 185 | -21% |
| **1Cs** | 363 | 397 | 9% | 233 | 185 | -21% |

As can be seen from Table S2 the value of D_c_/D_a_ < 1 indicates the formation of SSIPs in MeCN-D3 which is most pronounced for Cs with 0.77 decreasing by ascending the group 1 with 0.79 (Rb), 0.8 (K) and 0.9 for Na were the difference between Na and K is nonce more significantly higher than the difference between the heavier alkali metals K-Cs. This trend is reflected by the in Table 3 summarized values for the calculated molecular weight for the cationic and anionic fragment. The values suggest that both fragment moving freely in solution. As reported in C_6_D_6_ these values suggest that the equilibrium between molecular compounds with AM(crown)-PPh2 contact and SSIPs is shifted to the contact ion pairs.^[1]^

Figure S120. DOSY NMR spectrum of **1^Na^** in MeCN-D_3_.

Figure S121. DOSY NMR spectrum of **1^K^** in MeCN-D_3_.

Figure S122. DOSY NMR spectrum of **1^Rb^** in MeCN-D_3_.

Figure S123. DOSY NMR spectrum of **1^Cs^** in MeCN-D_3_.

Figure S124. Stacked ^31^P{^1^H} NMR spectra of **1^AM^** in MeCN-D3 at 300K. Purple (Cs), teal (Rb), green (K) and red (Na). Signals at 10 and 80 ppm belong to decomposition products of **1^AM^**.

# Section S5 – Kinetic Studies

For the kinetic studies allylanisole 3 (100 μmol) was used and first the catalyst concentration varied from 10-18 mol% in 2 mol% steps. NMR were measured every minute. Results are shown in Figures S125-S128. Second the substrate concentration was varied between 100-350 μmol in 50 μmol steps with constant catalyst concentration of 0.036 μmol/ml. Result are shown in Figures S129-S131.


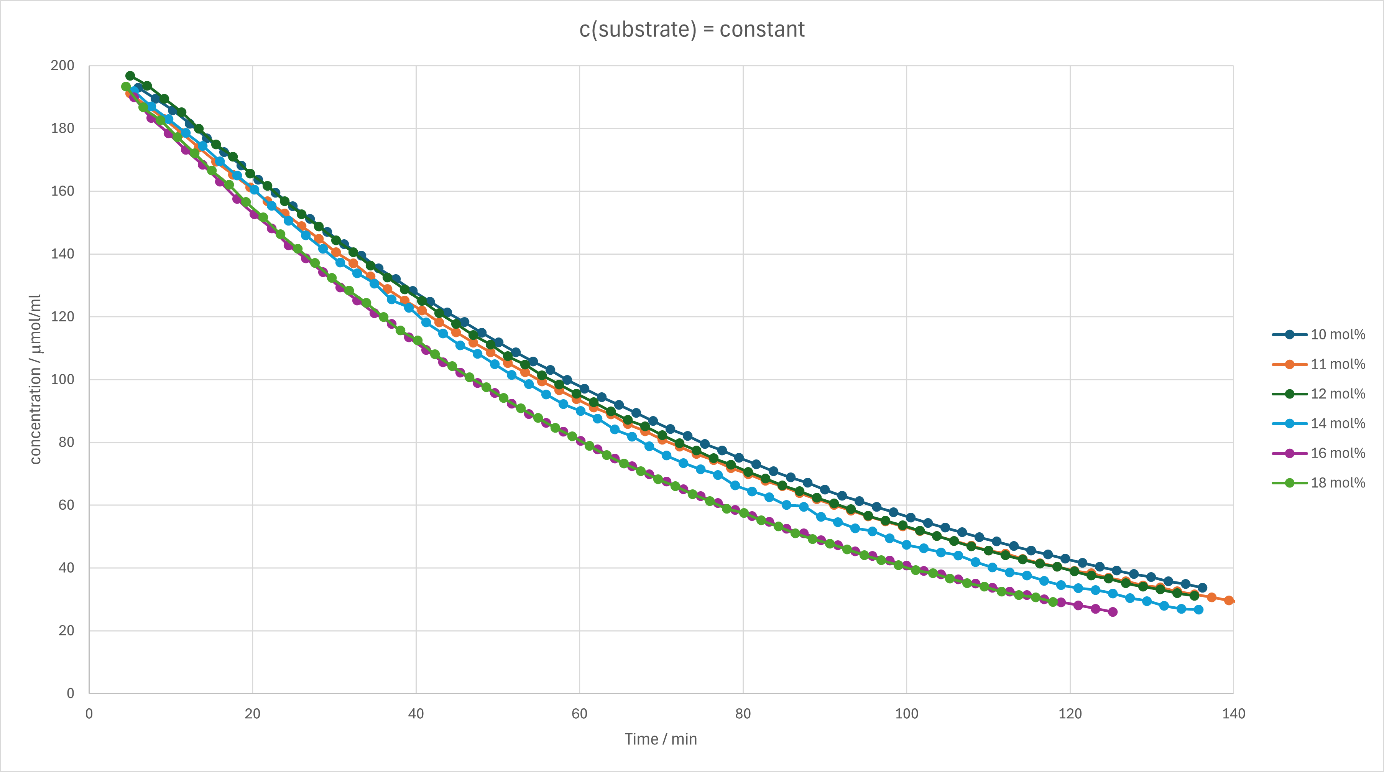


Figure S125. Plotted concentration vs reaction time in min.


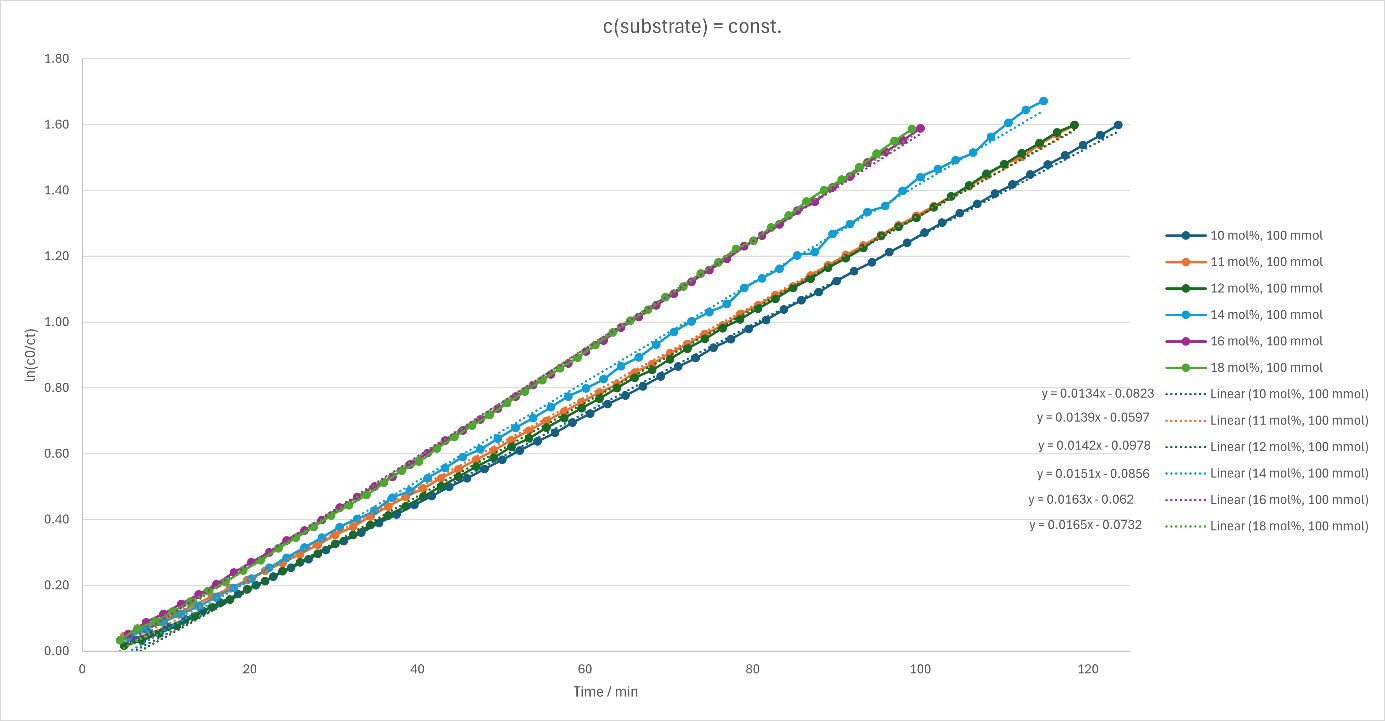


Figure S126. Plotted ln(c) vs reaction time with linear fit.


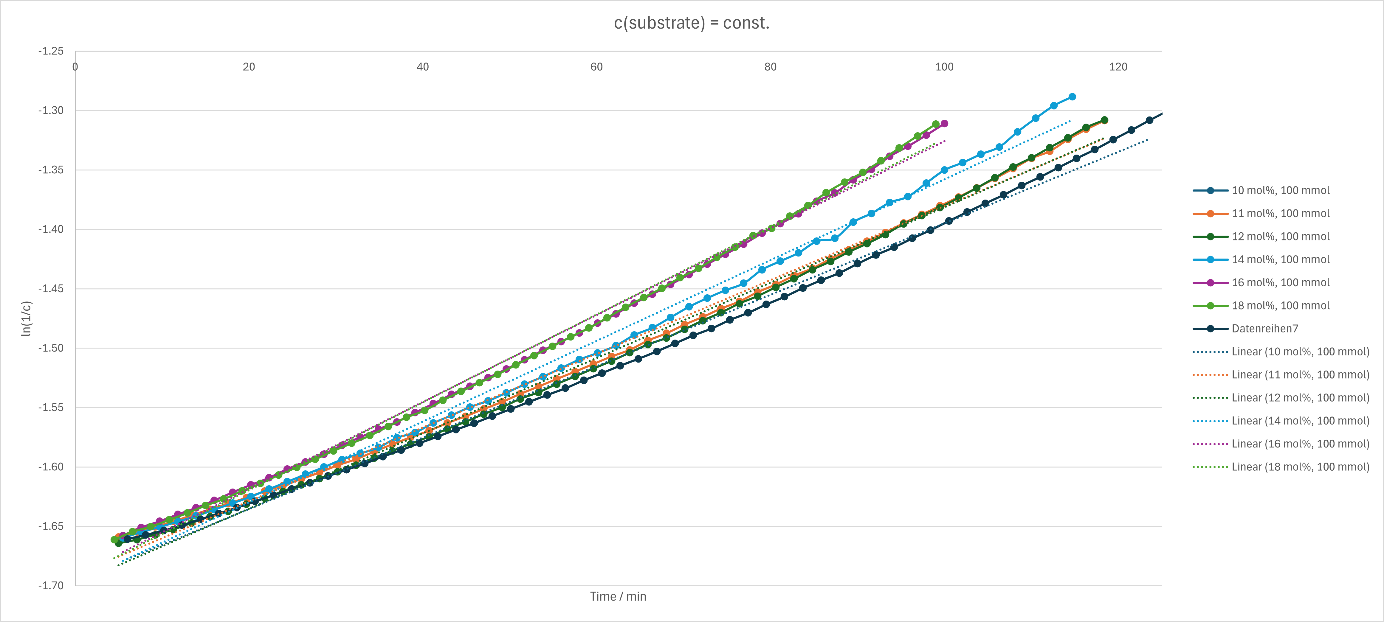


Figure S127. Plotted ln(1/c) vs reaction time with linear fit.


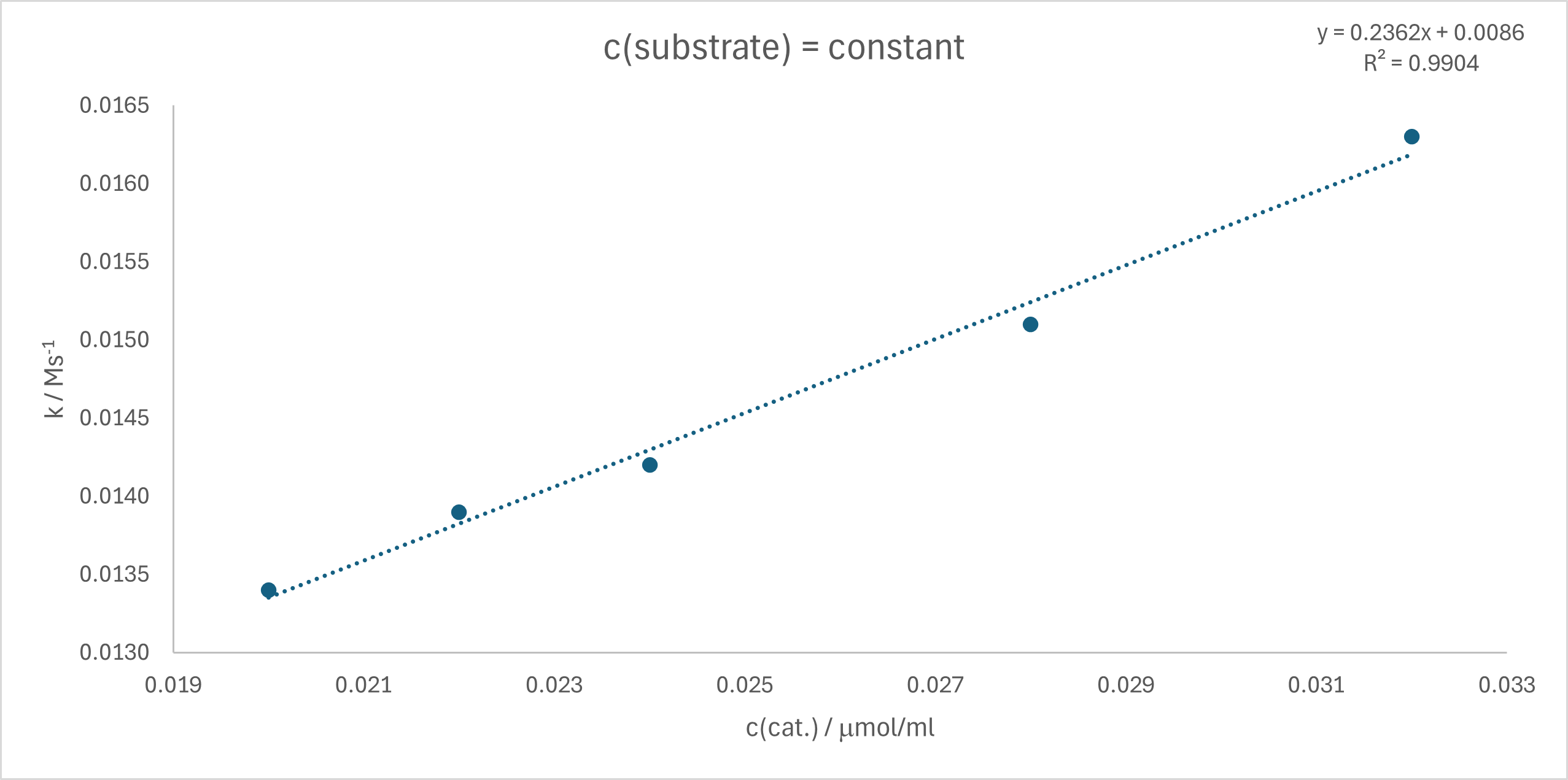


Figure S128. Plotted k vs catalyst concentration c(cat) resulting in 1^st^ order reaction for the catalyst.


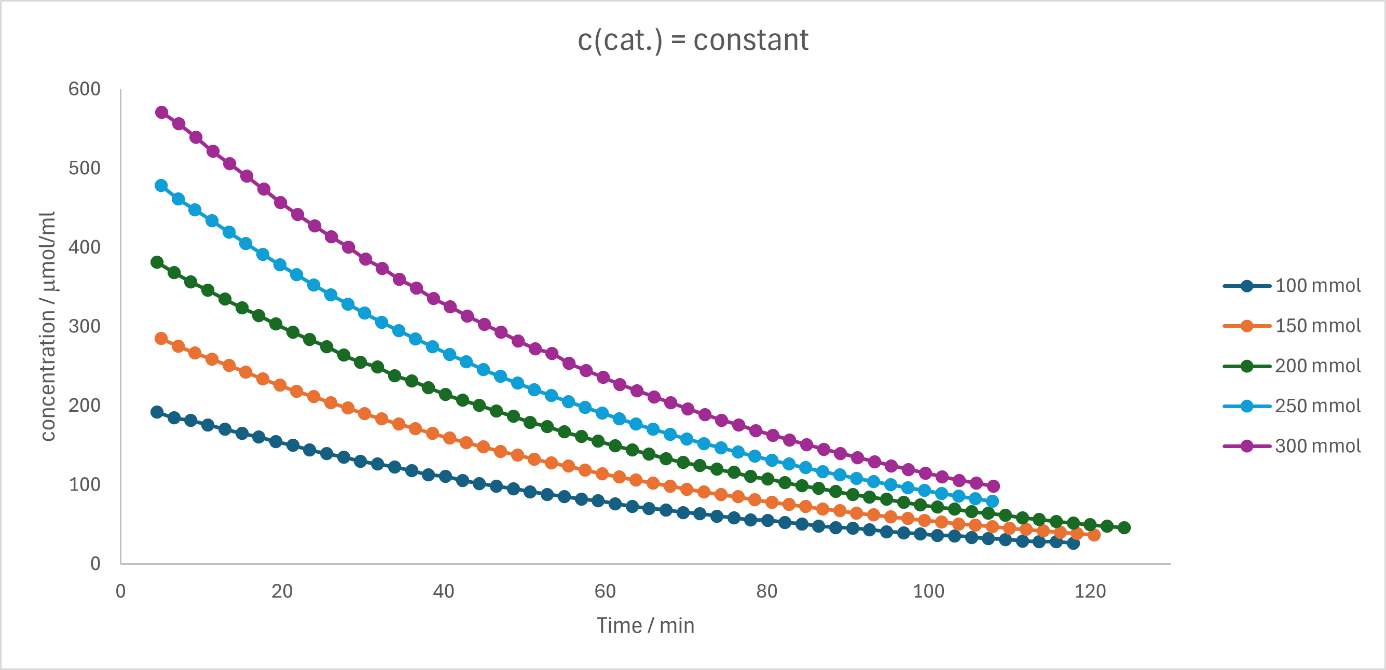


Figure S129. Plotted concentration vs reaction time in min.


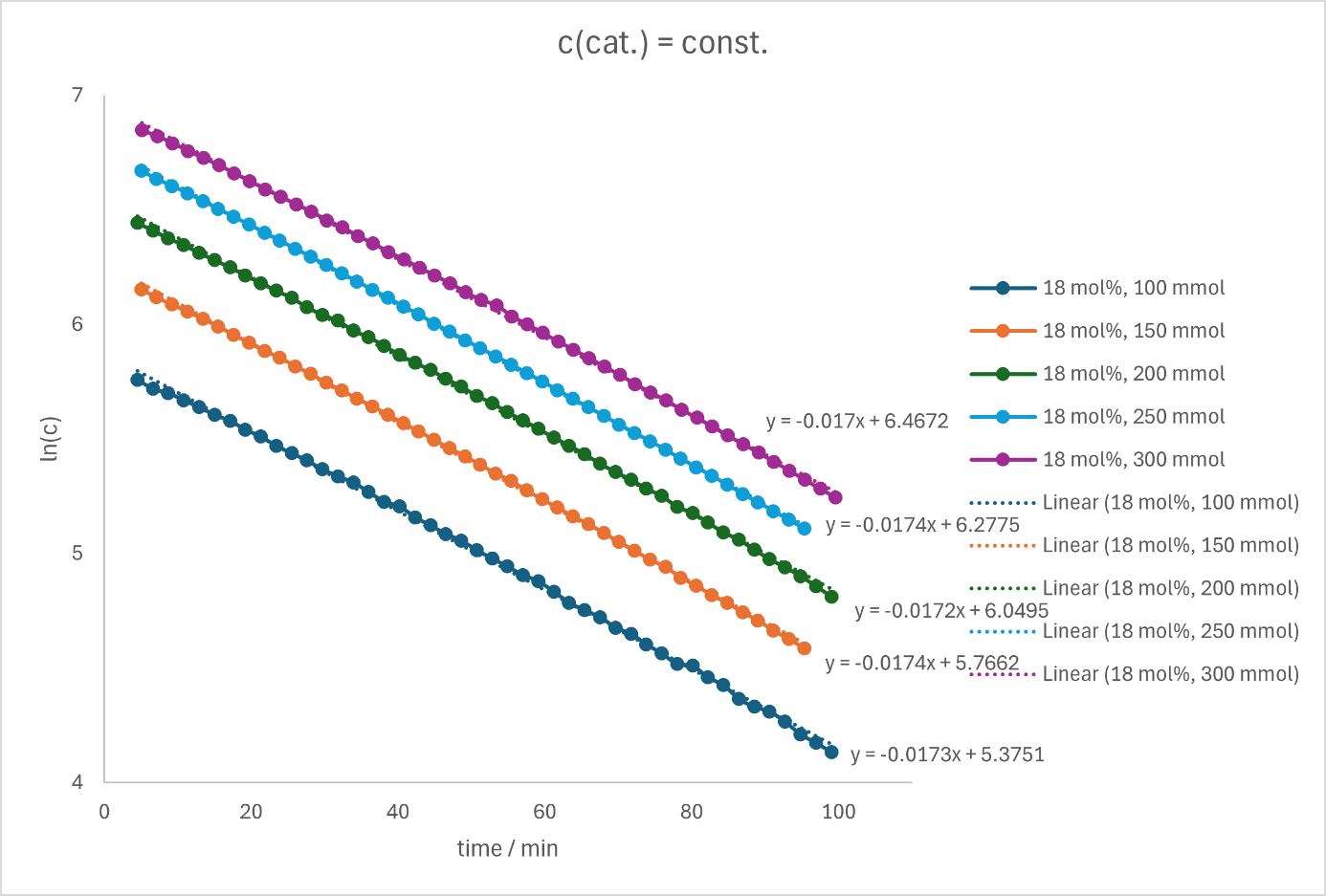


Figure S130. Plotted ln(1/c) vs reaction time with linear fit.


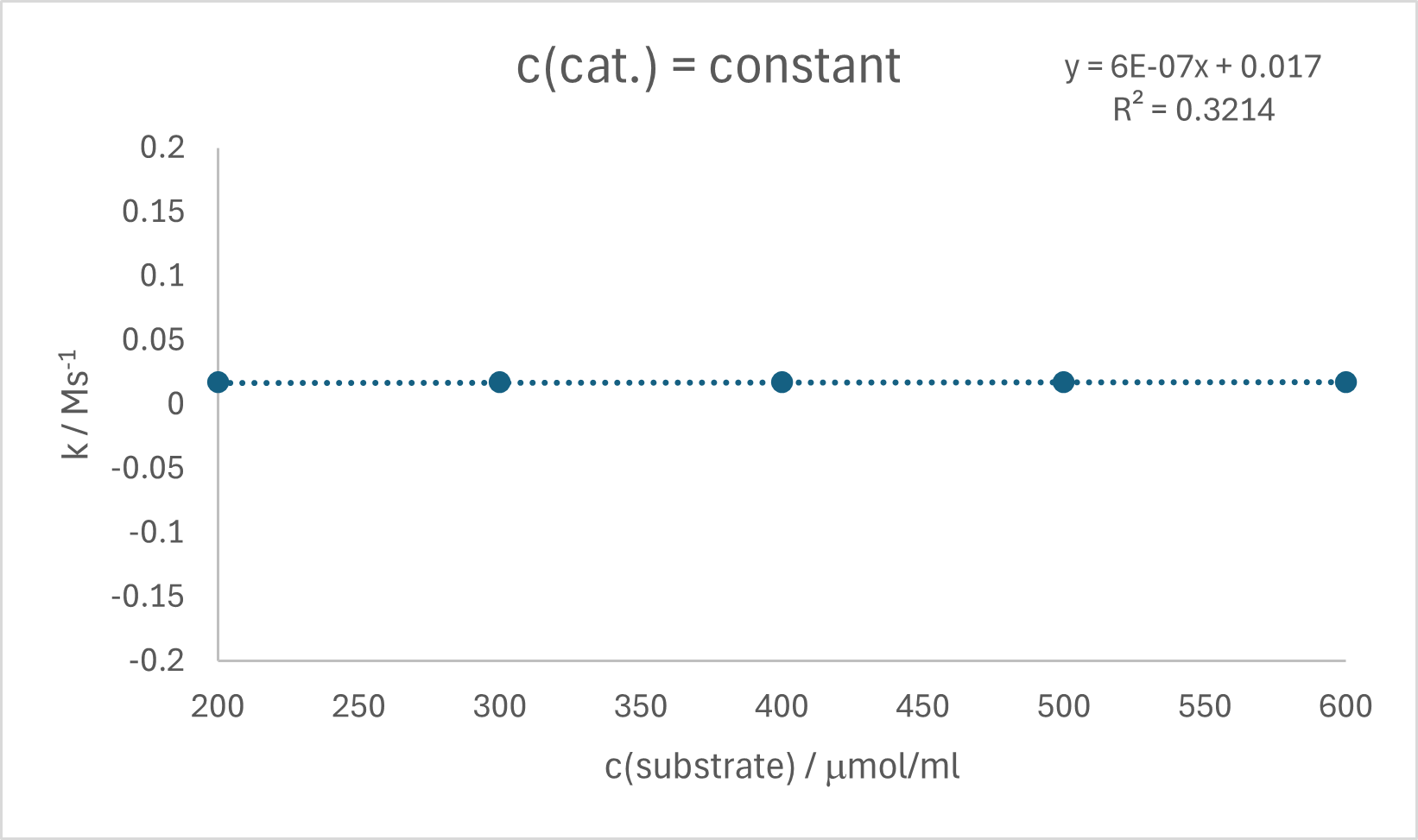


Figure S131. Plotted k vs substrate concentration c(substrate) resulting in zero order reaction for the substrate.


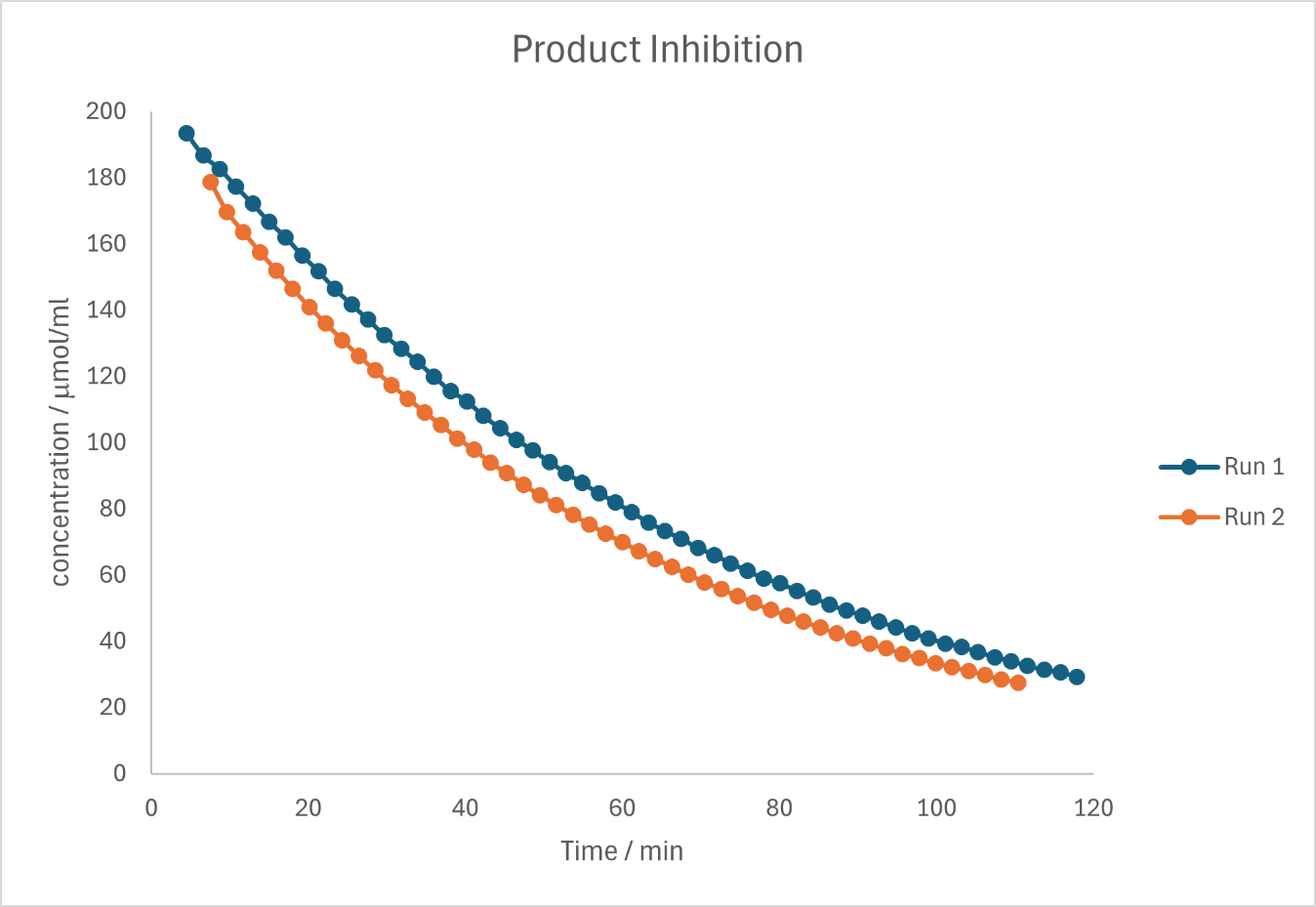


Figure S132. Plotted c vs time for two subsequent runs to check for product inhibition.

In order to get the experimental values for the activation energies the isomerisation of allylbenzene **2** was carried out at a 500 μmol scale for different temperature from 300-320 K for the catalysts **1^K^**, **1^Rb^** and **1^Cs^**. The resulting Arrhenius plots are displayed in Figures S133-S135


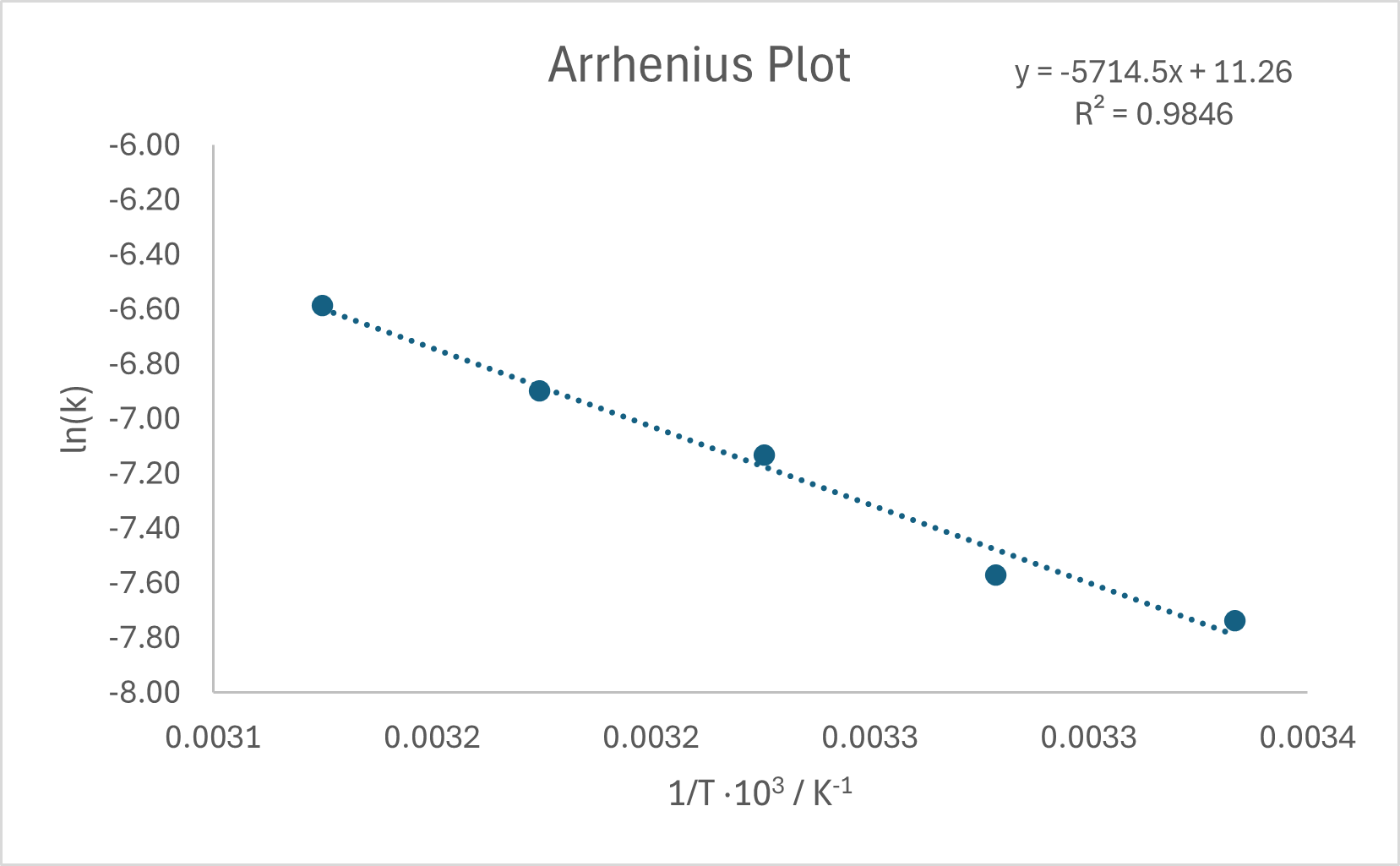


Figure S133. Arrhenius plot for **1^Cs^** resulting in an E_a_ = 11.4±0.60 kcal/mol.


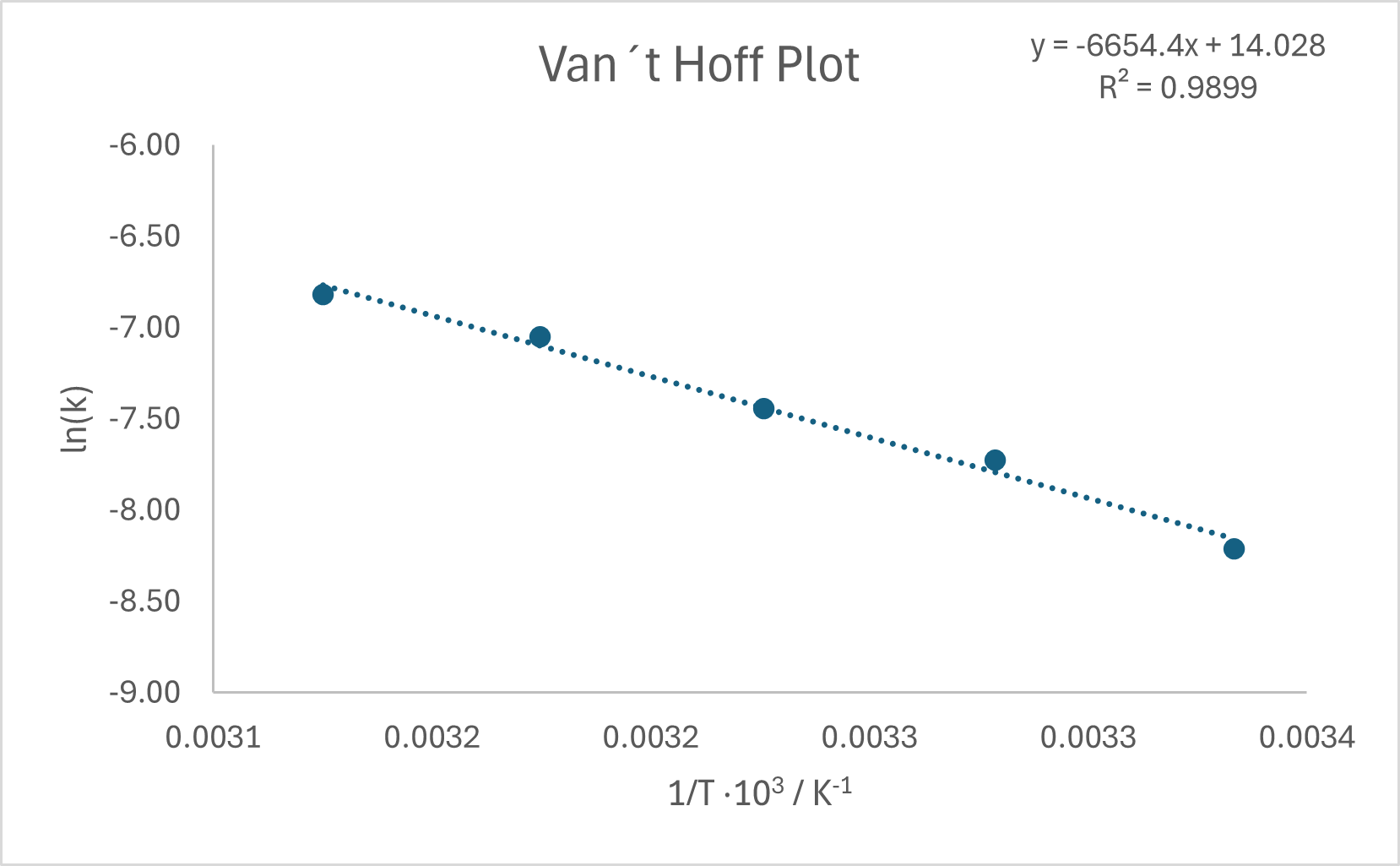


Figure S134. Arrhenius plot for **1^Rb^** resulting in an E_a_ = 13.2±0.64 kcal/mol.


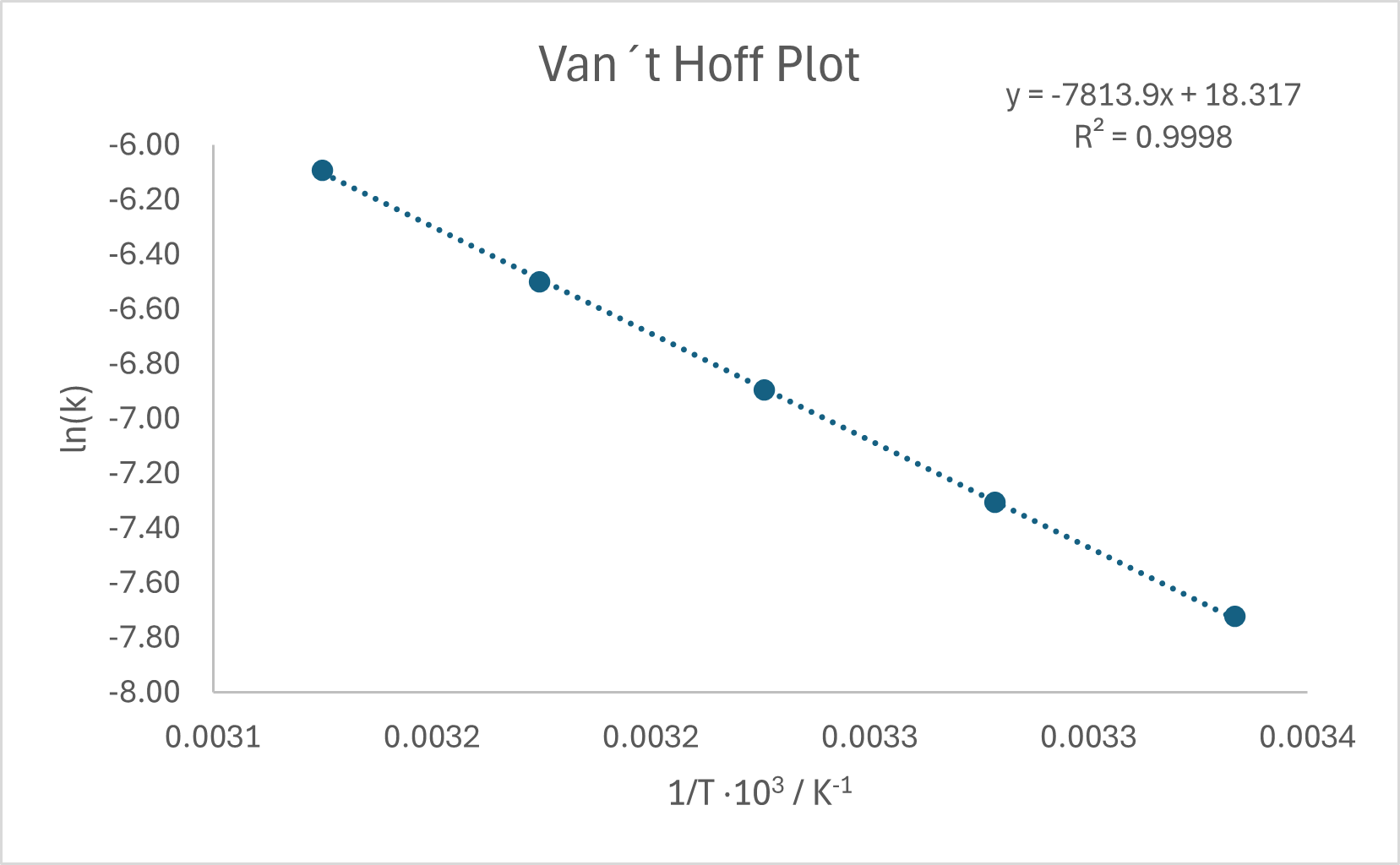


Figure S135. Arrhenius plot for **1^K^** resulting in an E_a_ = 15.5±0.12 kcal/mol.

# Section S6 – Crystallographic details

Crystallographic data for Cs(18-crown-6)-O-C_6_H_4_-(CH)_2_-CH_3_ was measured with a Rigaku Synergy-i instrument with monochromated Cu−Kα (λ = 1.54184 Å) radiation. The measured data was processed with the CrysAlisPro3 software package. The structures were solved in OLEX2 1.5^[12]^ by dual-space direct methods with SHELXT^[13]^, followed by full-matrix least-squares refinement using SHELXL.^[14]^ All non-hydrogen atoms were refined anisotropically. The contribution of the hydrogen atoms, in their calculated positions, was included in the refinement using a riding model. A full listing of atomic coordinates, bond lengths, angles and displacement parameters for all the structures has been deposited at the Cambridge Crystallographic Data Centre (CCDC 2492230)

Table S4. Crystal data and structure refinement data.

| \| Compound \| Cs(18-crown-6)-O-C_6_H_4_-(CH)_2_-CH_3_ \| \| --- \| --- \| \| CCDC# \| 2492230 \| \| Empirical formula \| Cs_2_O_8_C_30_H_42_ \| \| Formula weight \| 796.45 \| \| Temperature/K \| 159.98(10) \| \| Crystal system \| monoclinic \| \| Space group \| P2_1_/n \| \| a/Å \| 8.0900(2) \| \| b/Å \| 16.3625(3) \| \| c/Å \| 12.6245(3) \| \| α/° \| 90 \| \| β/° \| 100.480(2) \| \| γ/° \| 90 \| \| Volume/Å^3^ \| 1643.26(6) \| \| Z \| 2 \| \| ρ_calc_g/cm^3^ \| 1.61 \| \| μ/mm^‑1^ \| 17.667 \| \| F(000) \| 792 \| \| Crystal size/mm^3^ \| 0.424 × 0.134 × 0.07 \| \| Radiation \| Cu Kα (λ = 1.54184) \| \| 2Θ range for data collection/° \| 8.942 to 146.068 \| \| Reflections collected \| 11188 \| \| Independent reflections \| 3278 [R_int_ = 0.0380, R_sigma_ = 0.0285] \| \| Data/restraints/parameters \| 3278/0/182 \| \| Goodness-of-fit on F^2^ \| 1.044 \| \| Final R indexes [I>=2σ (I)] \| R_1_ = 0.0513, wR_2_ = 0.1537 \| \| Final R indexes [all data] \| R_1_ = 0.0536, wR_2_ = 0.1565 \| \| Largest diff. peak/hole / e Å^-3^ \| 1.55/-1.41 \| |
| --- | --- | --- | --- | --- | --- | --- | --- | --- | --- | --- | --- | --- | --- | --- | --- | --- | --- | --- | --- | --- | --- | --- | --- | --- | --- | --- | --- | --- | --- | --- | --- | --- | --- | --- | --- | --- | --- | --- | --- | --- | --- | --- | --- | --- | --- | --- | --- | --- | --- | --- | --- | --- | --- | --- | --- | --- |


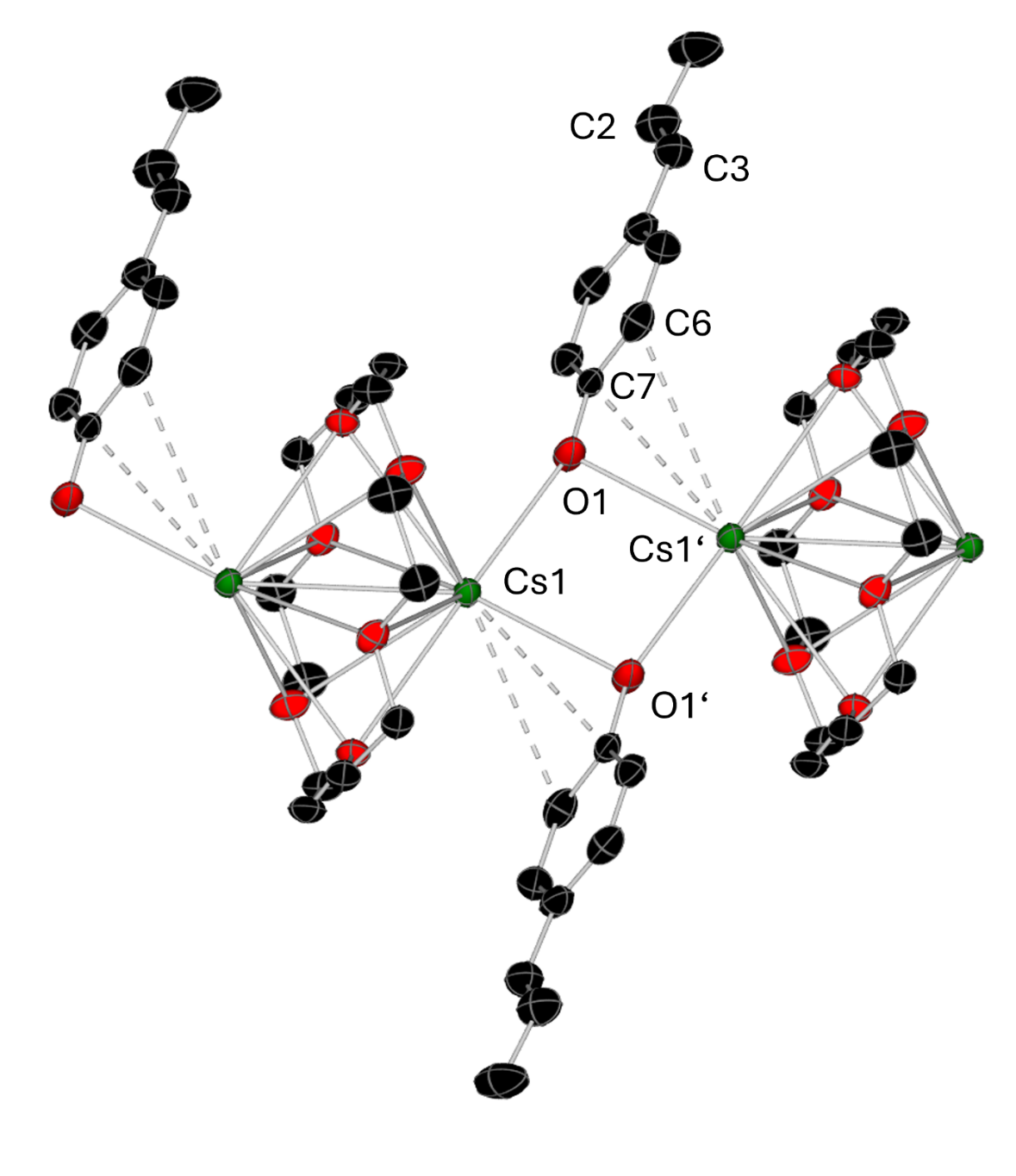


Figure S136. Molecular structure of Cs(18-crown-6)-O-C_6_H_4_-(CH)_2_-CH_3_ the solid state. Hydrogen atoms are omitted for clarity. Thermal ellipsoids have been drawn at 30% probability. Selected bond distances (Å) and angles (°): Cs1–O1 = 2.8602(37); Cs1–O1’ = 2.9852(40); Cs1–C7 = 3.5708(50); O1–C7 = 1.3038(59); C1–C2 = 1.4720(116); C2–C3 = 1.3330(98). O1–Cs1–O1 = 85.57(11).

# Section S7 – Computational Details

All calculations have been performed with the ORCA 5.0.4 or ORCA 6.1.0 program.^[15-21]^ Geometry optimizations of INT0-INT3 and TS1-TS2 were carried out using the functional BP86^[22-23]^ and the basis set def2-SVP^[24]^ including D3BJ dispersion correction^[25-26]^ and the SMD CPCM(benzene) solvent model^[27]^ as implemented in the ORCA Programme. For the catalysts, subtrate, product and the AM(crown)/PPh2 fragments the functional TPSSh^[28-29]^ and the triple-ζ basis set def2-TZVPP^[30]^ including D3BJ dispersion correction were used. All species were also characterized by frequency calculations and have positive definite Hessian matrices thus confirming that the computed structures are minima on the potential energy surface. Transition structures (TS’s) show only one negative eigenvalue in their diagonalized force constant matrices, and their associated eigenvectors were confirmed to correspond to the motion along the reaction coordinate under consideration using the Intrinsic Reaction Coordinate (IRC) method.^[31]^ In addition, the vibrational calculation provides the thermal Gibbs energy corrections by using the gas ideal-rigid-rotor-harmonic-oscillator approximation. Energies of INT0-INT3 and TS1-TS2 were refined by means of single point calculations employing the functional TPSSh^[28-29]^ combined with the much larger triple-ζ basis set def2-TZVPP^[30]^ including solvent effects. This level is referred to as CPCM(benzene)-TPSSh-D3BJ/def2-TZVPP//CPCM(benzene)-(RI)-BP86-D3BJ/def2-SVP. The xyz files of the optimized structures are deposited and available from: [https://doi.org/10.15129/7900f9e4-2b40-4b35-b814-e3506e7593fc](https://eur02.safelinks.protection.outlook.com/?url=https%3A%2F%2Fdoi.org%2F10.15129%2F7900f9e4-2b40-4b35-b814-e3506e7593fc&data=05%7C02%7Cr.e.mulvey%40strath.ac.uk%7C52366ab8b1b546e2170008de12fb779d%7C631e0763153347eba5cd0457bee5944e%7C0%7C0%7C638969068405573819%7CUnknown%7CTWFpbGZsb3d8eyJFbXB0eU1hcGkiOnRydWUsIlYiOiIwLjAuMDAwMCIsIlAiOiJXaW4zMiIsIkFOIjoiTWFpbCIsIldUIjoyfQ%3D%3D%7C0%7C%7C%7C&sdata=mCaB2EpE06N%2BL%2FJPcdblCl%2BpL1lcyOabjVDIJFR75OI%3D&reserved=0), reference number 315734319.


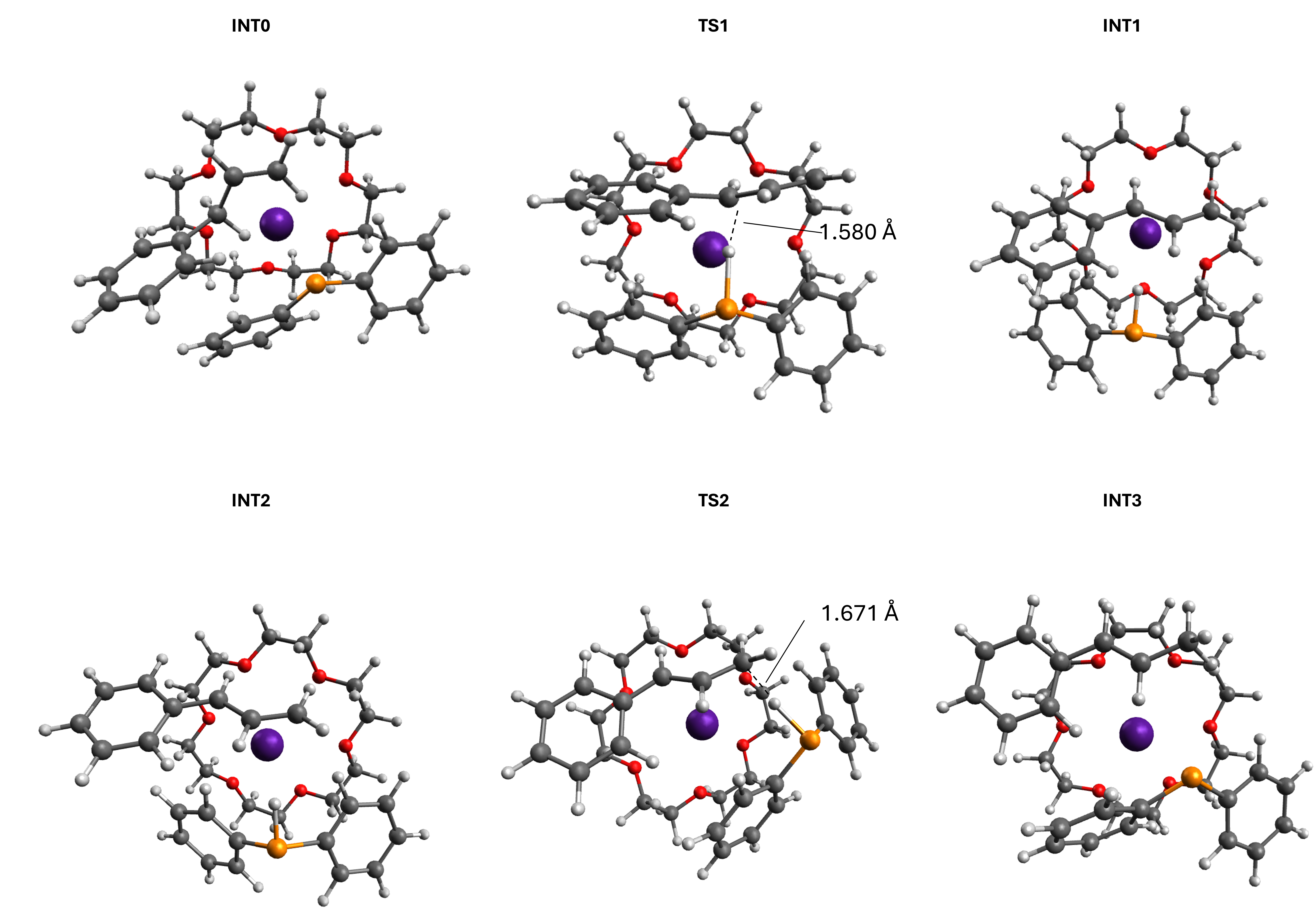


Figure S137. Calculated structures for the reaction pathway starting from CIP.


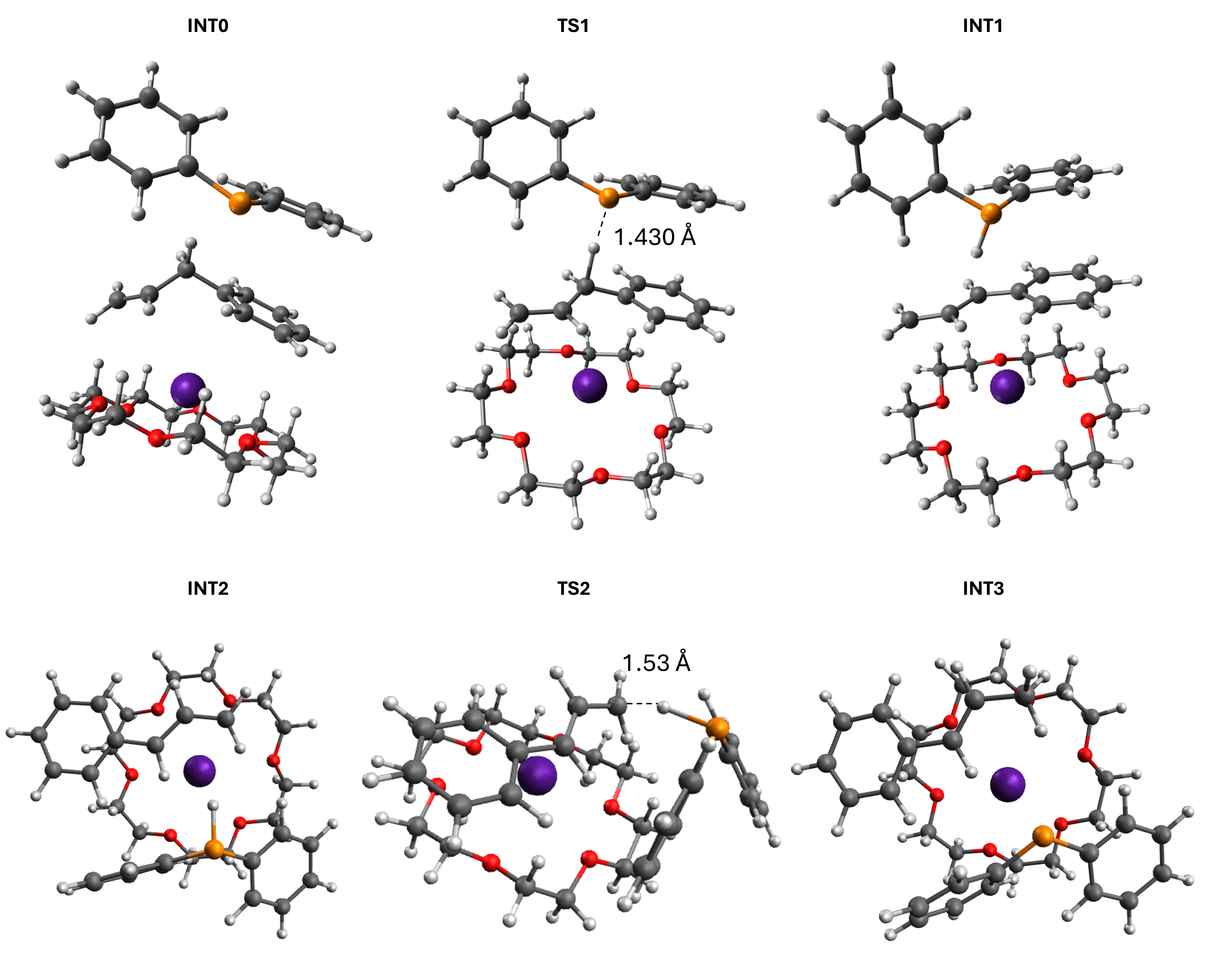


Figure S138. Calculated structures for the reaction pathway starting from SSIP.

# References

[1] F. Krämer, M. H. Crabbe, A. R. Kennedy, C. E. Weetman, I. Fernández, R. E. Mulvey, *Crown Ether Supported Alkali Metal Phosphides: Synthesis, Structures and Bonding, Chem. Eur. J.* **2025**, e02127.

[2] F. Krämer, M. H. Crabbe, I. Fernández, R. E. Mulvey, *Heavyweight Champion: Caesium Diorganophosphides Outperform Lighter Congeners in the Catalytic Hydrophosphination of Alkenes and Alkynes, Angew. Chem. Int. Ed.* **2025**, e202516376.

[3] Z. Rohlík, P. Holzhauser, J. Kotek, J. Rudovský, I. Němec, P. Hermann, I. Lukeš, *Synthesis and coordination properties of palladium(II) and platinum(II) complexes with phosphonated triphenylphosphine derivatives, J. Organomet. Chem.* **2006**, *691*, 2409-2423.

[4] V. L. Blair, M. A. Stevens, C. D. Thompson, *The importance of the Lewis base in lithium mediated metallation and bond cleavage reaction of allyl amines and allyl phosphines, Chem. Commun.* **2016**, *52*, 8111-8114.

[5] R. Neufeld, D. Stalke, *Accurate molecular weight determination of small molecules via DOSY-NMR by using external calibration curves with normalized diffusion coefficients, Chem. Sci.* **2015**, *6*, 3354-3364.

[6] S. Bachmann, B. Gernert, D. Stalke, *Solution structures of alkali metal cyclopentadienides in THF estimated by ECC-DOSY NMR-spectroscopy (incl. software), Chem. Commun.* **2016**, *52*, 12861-12864.

[7] S. Bachmann, R. Neufeld, M. Dzemski, D. Stalke, *New External Calibration Curves (ECCs) for the Estimation of Molecular Weights in Various Common NMR Solvents, Chem. Eur. J.* **2016**, *22*, 8462-8465.

[8] P. S. Pregosin, E. Martínez-Viviente, P. G. A. Kumar, *Diffusion and NOE NMR spectroscopy. Applications to problems related to coordination chemistry and homogeneous catalysis, Dalton Trans.* **2003**, 4007-4014.

[9] P. S. Pregosin, *Ion pairing using PGSE diffusion methods, Prog. Nucl. Magn. Reson. Spectrosc.* **2006**, *49*, 261-288.

[10] P. S. Pregosin, *NMR spectroscopy and ion pairing: Measuring and understanding how ions interact, Pure and Applied Chemistry* **2009**, *81*, 615-633.

[11] Z.-L. Xue, T. M. Cook, in *Comprehensive Inorganic Chemistry III (Third Edition)* (Eds.: J. Reedijk, K. R. Poeppelmeier), Elsevier, Oxford, **2023**, pp. 660-744.

[12] O. V. Dolomanov, L. J. Bourhis, R. J. Gildea, J. A. K. Howard, H. Puschmann, *OLEX2: a complete structure solution, refinement and analysis program, J. Appl. Crystallogr.* **2009**, *42*, 339-341.

[13] G. Sheldrick, *SHELXT - Integrated space-group and crystal-structure determination, Acta Crystallogr. Section A* **2015**, *71*, 3-8.

[14] G. Sheldrick, *Crystal structure refinement with SHELXL, Acta Crystallogr. Section C* **2015**, *71*, 3-8.

[15] F. Neese, *An improvement of the resolution of the identity approximation for the formation of the Coulomb matrix, J. Comput. Chem.* **2003**, *24*, 1740-1747.

[16] D. Bykov, P. Taras, I. Róbert, K. Simone, B. Ute, V. Edward, F. and Neese, *Efficient implementation of the analytic second derivatives of Hartree–Fock and hybrid DFT energies: a detailed analysis of different approximations, Molecular Physics* **2015**, *113*, 1961-1977.

[17] M. Garcia-Ratés, F. Neese, *Efficient implementation of the analytical second derivatives of hartree–fock and hybrid DFT energies within the framework of the conductor-like polarizable continuum model, J. Comput. Chem.* **2019**, *40*, 1816-1828.

[18] M. Garcia-Ratés, F. Neese, *Effect of the Solute Cavity on the Solvation Energy and its Derivatives within the Framework of the Gaussian Charge Scheme, J. Comput. Chem.* **2020**, *41*, 922-939.

[19] F. Neese, *Software update: The ORCA program system—Version 5.0, WIREs Comput. Mol. Sci.* **2022**, *12*, e1606.

[20] F. Neese, *The SHARK integral generation and digestion system, J. Comput. Chem.* **2023**, *44*, 381-396.

[21] F. Neese, *Software Update: The ORCA Program System—Version 6.0, WIREs Comput. Mol. Sci.* **2025**, *15*, e70019.

[22] J. P. Perdew, *Density-functional approximation for the correlation energy of the inhomogeneous electron gas, Phys. Rev. B* **1986**, *33*, 8822-8824.

[23] A. D. Becke, *Density-functional exchange-energy approximation with correct asymptotic behavior, Phys. Rev. A* **1988**, *38*, 3098-3100.

[24] F. Weigend, *Accurate Coulomb-fitting basis sets for H to Rn, Phys Chem Chem Phys* **2006**, *8*, 1057-1065.

[25] S. Grimme, J. Antony, S. Ehrlich, H. Krieg, *A consistent and accurate ab initio parametrization of density functional dispersion correction (DFT-D) for the 94 elements H-Pu, J. Chem. Phys.* **2010**, *132*, 154104.

[26] S. Grimme, S. Ehrlich, L. Goerigk, *Effect of the damping function in dispersion corrected density functional theory, J. Comput. Chem.* **2011**, *32*, 1456-1465.

[27] A. V. Marenich, C. J. Cramer, D. G. Truhlar, *Universal Solvation Model Based on Solute Electron Density and on a Continuum Model of the Solvent Defined by the Bulk Dielectric Constant and Atomic Surface Tensions, J. Phys. Chem. B* **2009**, *113*, 6378-6396.

[28] V. N. Staroverov, G. E. Scuseria, J. Tao, J. P. Perdew, *Comparative assessment of a new nonempirical density functional: Molecules and hydrogen-bonded complexes, J. Chem. Phys.* **2003**, *119*, 12129-12137.

[29] J. Tao, J. P. Perdew, V. N. Staroverov, G. E. Scuseria, *Climbing the Density Functional Ladder: Nonempirical Meta--Generalized Gradient Approximation Designed for Molecules and Solids, Phys. Rev. Lett.* **2003**, *91*, 146401.

[30] F. Weigend, R. Ahlrichs, *Balanced basis sets of split valence, triple zeta valence and quadruple zeta valence quality for H to Rn: Design and assessment of accuracy, Phys. Chem. Chem. Phys.* **2005**, *7*, 3297-3305.

[31] C. Gonzalez, H. B. Schlegel, *Reaction path following in mass-weighted internal coordinates, J. Phys. Chem.* **1990**, *94*, 5523-5527.
